# Evaluating the in vitro developmental toxicity potency of a series of petroleum substance extracts using new approach methodologies (NAMs)

**DOI:** 10.1007/s00204-023-03645-7

**Published:** 2023-12-12

**Authors:** Jing Fang, Ivonne M. C. M. Rietjens, Juan-Carlos Carrillo, Peter J. Boogaard, Lenny Kamelia

**Affiliations:** 1 Division of Toxicology, Wageningen University and Research, 6708 WE Wageningen, The Netherlands; 2 Shell Global Solutions International B.V, 2596 HR The Hague, The Netherlands

**Keywords:** Poorly refined petroleum substances, Highly refined petroleum substances, Petroleum-derived waxes, Polycyclic aromatic compounds, Developmental toxicity, New approach methodologies

## Abstract

The present study evaluates the in vitro developmental toxicity and the possible underlying mode of action of DMSO extracts of a series of highly complex petroleum substances in the mouse embryonic stem cell test (mEST), the zebrafish embryotoxicity test (ZET) and the aryl hydrocarbon receptor reporter gene assay (AhR CALUX assay). Results show that two out of sixteen samples tested, both being poorly refined products that may contain a substantial amount of 3- to 7-ring polycyclic aromatic compounds (PACs), induced sustained AhR activation in the AhR CALUX assay, and concentration-dependent developmental toxicity in both mEST and ZET. The other samples tested, representing highly refined petroleum substances and petroleum-derived waxes (containing typically a very low amount or no PACs at all), were negative in all assays applied, pointing to their inability to induce developmental toxicity in vitro. The refining processes applied during the production of highly refined petroleum products, such as solvent extraction and hydrotreatment which focus on the removal of undesired constituents, including 3- to 7-ring PACs, abolish the in vitro developmental toxicity. In conclusion, the obtained results support the hypothesis that 3- to 7-ring PACs are the primary inducers of the developmental toxicity induced by some (i.e., poorly refined) petroleum substances and that the observed effect is partially AhR-mediated.

**Supplementary Information:**

The online version contains supplementary material available at 10.1007/s00204-023-03645-7.

## Introduction

Petroleum substances are primarily produced by either atmospheric or vacuum distillation of crude oil, generating a range of products with various specifications and usage purposes (Mackerer et al. 2003; Concawe 2017). Petroleum substances are highly complex hydrocarbon materials, also known as UVCB substances (Unknown or Variable composition, Complex reaction products and Biological materials). The principle hydrocarbon classes present in petroleum substances are categorized into five groups, namely paraffinics (alkanes), isoparaffinics (isoalkanes), olefinics (alkenes), naphthenics (cycloalkanes), and aromatics (Feuston et al. 1994; Mackerer et al. 2003). Aromatics present in petroleum substances include monoaromatics, diaromatics and polyaromatics such as polycyclic aromatic compounds (PACs) (Feuston et al. 1994; Mackerer et al. 2003; Tsitou et al. 2015). When referring to ≥ 3 ring polyaromatics found in petroleum substances the term PACs is used to encompass three types of structures: naked/unsubstituted polycyclic aromatic hydrocarbons (PAHs), alkylated PAHs and heterocyclic aromatics (Feuston et al. 1994; Tsitou et al. 2015; Carrillo et al. 2019). The term PAH is often used as a surrogate for the entire PAC fraction (Andersson 2009; Kamelia et al. 2017; Wang et al. 2020, 2022).

**Fig. 1:**
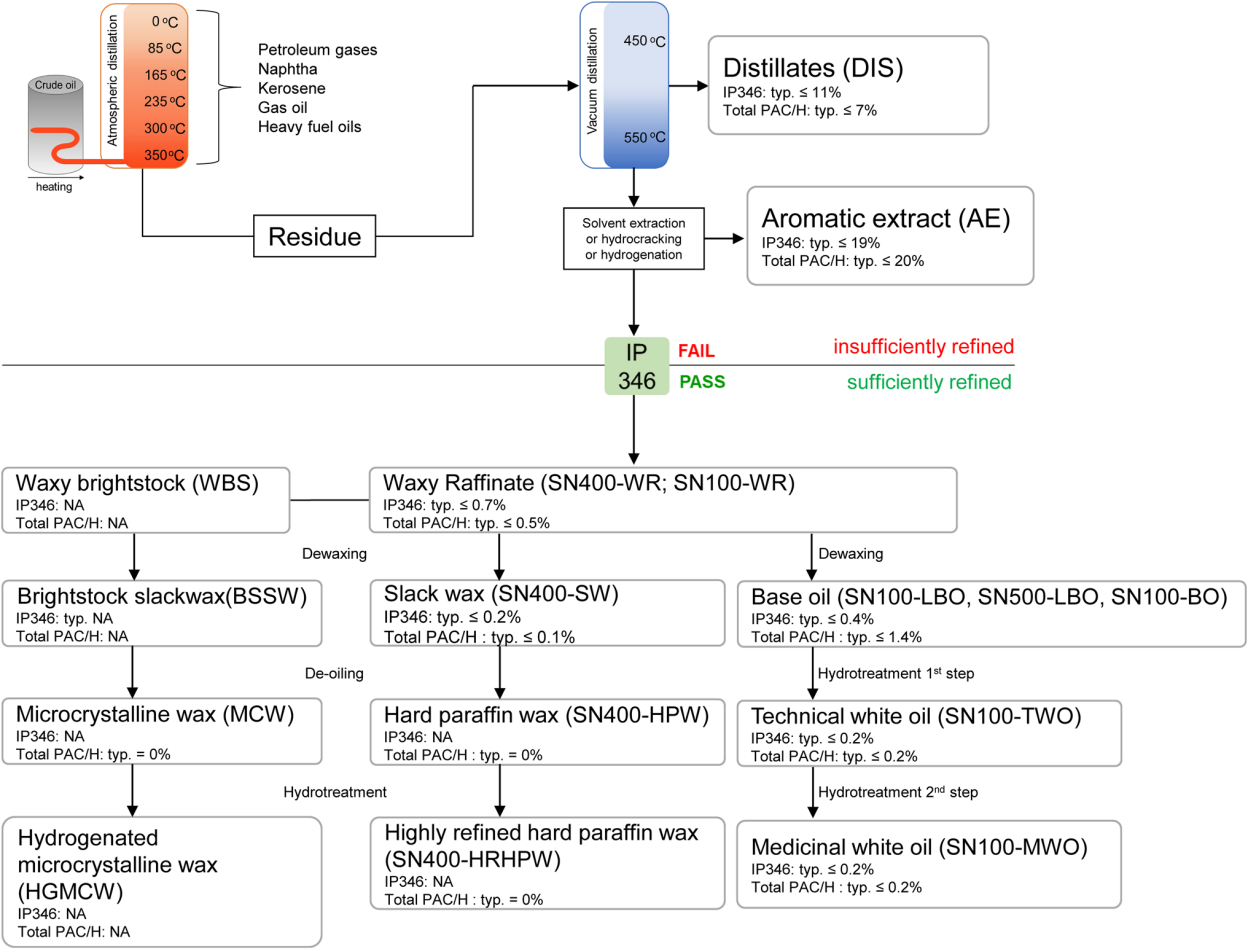
Flowchart of the manufacturing process of paraffinic petroleum products. IP346 is a gravimetric method based on a DMSO extraction to determine the total PAC in base oils (also known as mineral oils). The outcome of the IP346 test (cut-off: 3% w/w) can be used for the prediction of carcinogenicity potential for mineral oils (IP 1996). The typical values of the IP346 test and total PAC/H content of the various paraffinic petroleum products are obtained from the literature and are summarized in Supplementary Materials, Table S4. *Abbreviations. SN100-DIS*: SN100-Distillate, *SN100-AE*: SN100-Aromatic extract, *SN100-WR*: SN100-Waxy raffinate, *SN400-WR*: SN400-Waxy raffinate, *SN100-BO*: SN100-base oil, *SN100-LBO*: SN100-lubricating base oil, *SN500-LBO*: SN500-lubricating base oil, *SN100-TWO*: SN100-Techinical white oil, *SN100-MWO*: SN100-Medicinal white oil, *SN400-SW*: SN400-Slackwax, *SN400-HPW*: SN400-Hard paraffin wax, *SN400-HRHPW*: SN400-Highly refined hard paraffin wax, *WBS*: waxy brightstock, *BSSW*: brightstock slackwax, *MCW*: microcrystalline wax, *HGMCW*: hydrogenated microcrystalline wax

The type and quantity of aromatic constituents in petroleum substances vary depending on the source of the crude oil feedstock and the processing conditions applied during their manufacturing process (Mackerer et al. 2003; Tsitou et al. 2015; Carrillo et al. 2022). For example, aromatic extracts, obtained upon solvent extraction of distillates or the residue from a vacuum tower, contain a relatively high concentration of dimethyl sulfoxide (DMSO)-extractable aromatics (6.6–20.3%, mostly 3- to 7-ring PACs) (HPV 2012; Carrillo et al. 2021). Whereas, highly refined petroleum products, such as highly refined base oil (HRBO) and petroleum derived-waxes, obtained upon dewaxing, deoiling and/or hydrotreatment of base oil process, contain mainly highly alkylated 1- to 2-ring aromatics because the hazardous 3- to 7-ring aromatic compounds in these products are removed during the refinement (Fig. 1) (Mackerer et al. 2003; Carrillo et al. 2019, 2021, 2022; Pirow et al. 2019). At comparable refining conditions, the level of DMSO extractable-aromatics may be used as a surrogate of the amount of PAC content of a substance and highly refined substances yield less DMSO extractables. Such highly refined petroleum substances and petroleum-derived-waxes are generally considered as inert and of high purity, thus are used for various applications including cosmetic production, pharmaceutical production, and food packaging materials (Pirow et al. 2019).

The developmental toxicity which is observed in animal studies with heavier petroleum substances, such as heavy fuel oil and aromatic extracts, is associated with the presence of polyaromatics (mainly 3- to 7-ring PACs) in these substances (Feuston et al. 1989, 1994; Murray et al. 2013; Tsitou et al. 2015). Recent studies (Kamelia et al. 2017, 2018, 2019a, 2019b, 2021) showed that the developmental toxicity of PAC-containing petroleum substances was successfully evaluated using a battery of in vitro alternative assays, comprising the mouse embryonic stem cell test (mEST), the zebrafish embryotoxicity test (ZET), and the aryl hydrocarbon receptor CALUX reporter gene assay (AhR CALUX assay). It was demonstrated that the developmental toxicity induced by DMSO extracts of some petroleum substances was proportional to the level of 3- to 7-ring PACs present in these extracts, in agreement with the aforementioned in vivo observations. Also, it was shown that the observed in vitro developmental toxicity in both mEST and ZET was partially AhR-mediated (Kamelia et al. 2017, 2018, 2019a, 2019b, 2021). Since the highly refined petroleum products mainly contain 1- to 2-ring aromatics, and contain low or no levels of 3–7 ring PACs (Carrillo et al. 2022) thus they are not expected to induce developmental toxicity.

The present study aims to extend the applicability domain of the test battery beyond the previously tested petroleum samples by evaluating the in vitro developmental toxicity and the underlying mode of action to induce developmental toxicity of an additional series of petroleum substances extracts using the AhR reporter gene assay, mEST and ZET. In total, sixteen samples with varying degrees of refinement were tested in the present study (Fig. 1), which include distillates, aromatic extracts, highly refined petroleum substances, and petroleum substances-derived waxes. Two out of sixteen selected samples were a distillate and an aromatic extract, which are not extensively refined, thus containing relatively high amounts of 3- to 7-ring PACs. The other fourteen samples were further treated by various refinement processes to remove the undesired 3- to 7-ring PACs, thus they typically contain either low amounts of biologically active aromatics or none at all. The results are expected to broaden the applicability domain of the new approach methodologies (NAMs) previously applied (Kamelia et al. 2017, 2018, 2019a, 2019b, 2021) and allow the assessment of the developmental toxicity of not only petroleum substances with relatively high PAC levels but also of highly refined petroleum substances and petroleum-derived waxes with low level or devoid of PACs.

## Materials and methods

### Test compound

Benzo[a]pyrene (BaP; CAS no. CAS No. 50–32–8), 5-fluorouracil (CAS no. 51–21-8) and 3,4-dichloroaniline (CAS no. 95–76-1) were purchased from Sigma-Aldrich (Zwijndrecht, The Netherlands). All stocks and dilutions of test compounds were prepared in dimethyl sulfoxide (DMSO) (Merck, Darmstadt, Germany).

DMSO extracts of the sixteen complex petroleum substances (Fig. 1) were tested, including a distillate (sample SN100-DIS, CAS No. 64741–50-0), an aromatic extract (sample SN100-AE, CAS No. 64742–05-8), waxy raffinates (samples SN100-WR and SN400-WR; CAS No. 64741–89-5), base oils (sample SN100-LBO, CAS. No 64742–65-0; sample SN500-LBO, CAS. No 64742–65-0; sample SN100-BO, CAS No. 64742–56-9), a technical white oil (sample SN100-TWO, CAS No. 8042–47-5), a medicinal white oil (sample SN100-MWO, CAS No. 8042–47-5), a slackwax (sample SN400-SW, CAS No.64742–61-6), a hard paraffin wax (sample SN400-HPW, CAS No. 8002–74-2), a highly refined hard paraffin wax (sample SN400-HPHPW, CAS No.64742–51-4), a waxy brightstock (sample WBS, CAS No. not available), a brightstock slackwax (sample BSSW, CAS No. not available), a microcrystalline wax (sample MCW, CAS No. 63231–60-7), and a hydrogenated microcrystalline wax (sample HGMCW, CAS No. 64742–60-5).

### DMSO extraction procedure

To generate DMSO extracts of the substances tested, the method previously described by Roy et al. 1988, McKee et al. 2014 and Kamelia et al. 2017 was applied. Briefly, 4.0 g of each test material was dissolved in 10 ml cyclohexane (CAS No. 110–82-7, Sigma-Aldrich) and then extracted twice with 10 ml DMSO. The extract was collected in a capped brown glass vial and stored at 4 °C until use.

### Aryl hydrocarbon (AhR) CALUX reporter gene assay

In the present study, the H4IIE.luc AhR CALUX reporter gene assay was performed essentially as described by Kamelia et al. (2018).

#### Cell line and cell culture conditions

The stably transfected rat hepatoma cell line (H4IIE.luc) was used for the AhR CALUX assay (Aarts et al. 1995). H4IIE.luc cells were cultured in 75 cm^2^ polystyrene cell culture flasks (Corning, The Netherlands) with Minimum Essential Medium (MEM) alpha medium (Gibco, Paisley, UK, catalog no. 2256–021) which was supplemented with 10% (v/v) fetal bovine serum (FBS; Sigma-Aldrich, The Netherlands) at 37 °C with 5% CO_2_ in a humidified atmosphere. All cells were routinely sub-cultured using 0.05% trypsin–EDTA (Gibco) for every 2 to 3 days.

#### AhR CALUX reporter gene assay

In the present study, the H4IIE.luc AhR CALUX reporter gene assay was performed to evaluate the AhR-mediated activity of the test compounds. First, H4IIE.luc cells were washed with phosphate-buffered saline (PBS; Gibco) and trypsinized with 0.05% trypsin–EDTA when reaching 80–90% confluency. Then, 100 μl of cell suspension at a density of 3 × 10^5^ cells/ml was seeded into each of the 60-inner wells of white 96-well plates (Greiner Bio-one, Frickenhausen, Germany); the 36-outer wells of the same white 96-well plate were filled with 200 µl PBS to limit evaporation from the inner wells. Cells were incubated for 24 h at 37 °C with 5% CO_2_. After the incubation, cells were exposed to an exposure medium for either 6 h or 24 h, in triplicate. The exposure medium was prepared by adding 400 times concentrated test compounds dissolved in DMSO into pre-conditioned medium. Pre-conditioned medium was the growth medium harvested from cells which have been already cultured for 16 – 24 h. The use of pre-conditioned medium helps to reduce the high background luciferase signal induced by tryptophan products present in fresh medium (Hamers et al. 2000). After 6 h or 24 h exposure, cells were washed with 100 μl ½ PBS (PBS: nano-pure water = 1:1) and lysed with 30 μl low salt buffer (LSB; 10 mM Tris (Sigma-Aldrich), 2 mM dithiothreitol (DTT, Sigma-Aldrich), and 2 mM 1, 2-diaminocyclohexanete triacetic acid monohydrate (Sigma-Aldrich); pH 7.8). Afterwards, plates were placed on ice for at least 15 min and frozen at − 80 °C for at least 120 min before luminescence measurement.

For the luminescence measurement, a luminometer (Glomax-Multi Detection System, Promega, California) was used. Before measurement, plates were thawed at room temperature for 1 h and placed on a plate shaker for around 5 min. Then, 100 μl flash-mix solution was added to each well. The flash-mix solution contained 20 mM tricine (Sigma-Aldrich), 1.07 mM (MgCO_3_)_4_ Mg (OH)_2_·5H_2_O (Sigma-Aldrich), 2.67 mM magnesium sulfate (MgSO_4_, Merck), 0.1 mM ethylenedinitrilotetraacetic acid disodium salt dihydrate (Merck), 2 mM DTT (Sigma-Aldrich), 0.47 mM D-luciferin (Synchem UG & Co. KG, Felsberg, Germany), and 5 mM adenosine-5-triphosphate (Duchefa Biochemie bv, Haarlem, The Netherlands).

All test compounds were tested in the AhR CALUX assay at concentrations up to 5 μg/ml, and the final concentration of the solvent DMSO was kept at 0.25% (v/v). A full concentration–response curve of the positive control BaP up to the concentration of 1.26 × 10^−1^ μg/ml was included in each independent experiment. Three independent experiments were conducted for each test compound.

### Mouse embryonic stem cell test (mEST)

The ES-D3 cell viability and differentiation assays of the mEST were performed as described by Kamelia et al. (2017, 2020).

#### Cell line and cell culture conditions

The pluripotent mouse ES-D3 cell line (ATCC, Wesel, Germany) was used for the mEST. ES-D3 cells were cultured in 25 cm^2^ polystyrene cell culture flasks (Corning, The Netherlands). The flasks were pre-coated with 0.1% (w/v) gelatine for at least 1 h before use. ES-D3 cells were cultured in mouse ES cell basal medium (ATCC, USA) supplemented with 15% FBS, ES cell qualified (ATCC, USA), 50 U/ml penicillin with 50 µg/ml streptomycin (Invitrogen), 2 mM L-glutamine (Invitrogen), 0.5% non-essential amino acids (NEEA; Invitrogen), and 0.1 mM 2-mercaptoethanol (Gibco) at 37 °C with 5% CO_2_ in a humidified atmosphere. Cells were kept undifferentiated by the addition of 1000 U/mL murine Leukemia Inhibiting Factor (mLIF; Sigma-Aldrich). The ES-D3 cells were routinely sub-cultured every 2–3 days using a non-enzymatic cell dissociation solution (Sigma-Aldrich).

#### ES-D3 cell viability assay of the mEST

The water-soluble tetrazolium (WST-1) assay was performed to determine the cell viability following exposure to test compounds. The principle of the assay is based on the ability of metabolically active cells to convert the tetrazolium salt WST-1 into formazan dye by mitochondrial dehydrogenase (Ngamwongsatit et al. 2008). First, 100 µl ES-D3 cell solution at a density of 2 × 10^5^ cells/ml (1-day exposure) or 1 × 10^4^ cells/ml (5 days exposure) was seeded into the 60-inner wells of 96-well plates (Greiner Bio-One) in the absence of mLIF. Then, cells were incubated for 24 h at 37 °C with 5% CO_2_ in a humidified atmosphere to allow cell adherence. After the incubation, ES-D3 cells were exposed to increasing concentrations of test compounds by adding 100 µl exposure medium to each well. The exposure medium was prepared by mixing the 400 times concentrated test compounds in DMSO into ES-D3 growth medium (without mLIF). All test compounds were tested in the ES-D3 cell viability assay at concentrations up to 250 μg/ml and the final concentration of the solvent DMSO was kept at 0.25% (v/v). The cells were exposed to each concentration of the test compounds in triplicate and incubated for 1 or 5 days at 37 °C with 5% CO_2_. After the incubation for 1 or 5 days, 10 μl of WST-1 reagent (Roche, Woerden, The Netherlands) was added into each well, then cells were further incubated for 3 h at 37 °C with 5% CO_2_. Finally, the absorbance of the formed formazan was measured at 440 nm (background at 620 nm) using a SpectraMax iD3 (Molecular Devices, San Jose, USA). Three independent experiments were conducted for each test compound.

#### ES-D3 cell differentiation assay of the mEST

The ES-D3 cell differentiation assay of the mEST was performed as previously described by Kamelia et al. 2017. On day 0, droplets of 20 μl ES-D3 cell solution (3.75 × 10^4^ cells/mL) with or without test compounds were placed between the well borders on the inner side of the lid of a 96-well plate. To create an optimal humidity and prevent evaporation of the hanging drops, each well of the 96-well plates was filled with 250 μl PBS. Sterile caps of Eppendorf tubes were placed in the corner of the plates to prevent direct contact of the hanging drops with the plate, and the plate was then sealed with Micropore Tape (3 M, Neuss, Germany). After 72 h incubation at 37 °C with 5% CO_2_ in a humidified atmosphere, or on day 3, the formed embryoid bodies (EBs) were collected and transferred to non-tissue culture-treated petri dishes (diameter 6 cm, Greiner), containing 5 ml of medium with or without test compounds. The petri dishes were then incubated for another 48 h (2 days) at 37 °C with 5% CO_2_. On day 5, the EBs were transferred to a 24-well plate (Corning), 1 EB per well, containing 1 ml of medium with a test compound. The 24-well plates were incubated for 5 days at 37 °C with 5% CO_2_. On day 10, the number of wells containing contracting cardiomyocytes was determined by visual inspection using a light microscope. 0.25% v/v DMSO (solvent control) and 0.3 μM 5-fluorouracil (positive control) were included in each independent experiment. The ES-D3 cell differentiation assay was considered valid if the solvent control in each experiment had at least 21 out of 24 wells that contained contracting cardiomyocytes. Three independent experiments were conducted for each test compound.

### Zebrafish Embryotoxicity Test (ZET)

In the present study, the ZET was performed essentially as described by Kamelia et al. (2019a). Fertilized eggs of wild-type zebrafish (*Danio rerio*) AB line were purchased from the research facility Carus, at Wageningen University and Research, The Netherlands. The ZET was performed as previously described (Kamelia et al. 2019a). Briefly, the zebrafish embryos were exposed to the test compounds at 4–5 h post fertilization (hpf). 24-well plates (Greiner Bio-One) were used for the exposure: twenty wells were used for exposure to two concentrations of test compounds (ten wells for each concentration) and the other four wells were used for the internal plate control. The exposure medium was prepared by mixing 400 times concentrated DMSO stock solutions of test compounds with egg water. The egg water was prepared by adding 10 ml 100-times egg water stock solution (3 g sea salt (Tropic marine, Wartenberg, Germany) in 500 ml demineralized water) to 990 ml demineralized water. All test compounds were tested in the ZET at concentrations up to 250 μg/ml, except for SN100-AE, which was tested up to 25 μg/ml because at such concentration this test substance already induced 100% cumulative mortality at 96 hpf. The exposure medium was transferred (2 ml/well) into 20 wells of the 24-well plate, and 2 ml egg water was added into each of the 4 remaining wells for the internal plate control. One zebrafish embryo was transferred to each well of the 24-well plate (1 embryo/well). The plates were sealed with self-adhesive film covers (Greiner) to prevent evaporation and were incubated at 26 °C with a photoperiod of 14 h light:10 h dark. Three controls were included in each independent experiment: solvent control (0.25% v/v DMSO), positive control (4 μg/ml 3,4-dichloroaniline) (Sigma-Aldrich), and negative control (egg water only). Embryos were scored daily using an inverted microscope until 96 hpf for embryo lethality and developmental abnormalities, based on the extended general morphological scoring (extended-GMS) system described by Beekhuijzen et al. 2015. This extended-GMS system includes two parts: 1) general development of zebrafish embryos (GMS), and 2) dysmorphogenic endpoints. The GMS consists of twelve endpoints i.e., detachment of tail, somite formation, eye development and pigmentation, movement, circulation, presence of heartbeat, pectoral fins, pigmentation of head and body, pigmentation of the tail, hatching, presence of protruding mouth, and yolk extension. The dysmorphogenic endpoint scoring consisted of six endpoints i.e., yolk sac edema, pericardial sac edema, malformation of the tail, deformed body shape, malformation of the head and jaw, and malformation of the sacculi/otoliths (Supplementary material 1). Any deviation from normal development of zebrafish embryos results in a lower extended-GMS score, corresponding to a certain extent of developmental retardation. The ZET was considered valid if the following was observed (at 96 hpf): (1) ≤ 1 dead embryo (out of 4) in the internal plate control of the exposed plate; (2) ≤ 1 dead embryos (out of 10) in the negative control plate; (3) ≤ 1 dead embryos (out of 10) in the solvent control plate; (4) ≤ 7 live embryos (out of 10) in the positive control plate. At least three independent experiments were conducted for each test compound.

### Data analysis

Figures of concentration–response curves in the AhR CALUX assay, ZET and mEST were generated using GraphPad Prism 5.0 (California, USA). Data were fitted into sigmoid concentration–response curves using non-linear regression analysis with three parameters (i.e., the top, bottom and middle) equal to a Hill slope (or slope factor) of 1.0. Results were presented as mean percentage of induction factor (AhR CALUX assay) ± standard error of the mean (SEM), % of cell viability (ES-D3 cell viability assay) ± SEM, fraction of total % (ES-D3 cell differentiation assay) ± SEM, or response % (ZET) ± SEM from at least three independent experiments. It should be noted that except for SN100-DIS, SN100-AE, SN100-LBO and SN500-LBO, other petroleum samples applied induced no effect at the highest concentration i.e., 250 μg/ml, in both mEST and ZET, thus they were only tested at three concentrations: 0.5 (low), 125 (middle) and 250 (high) μg/ml in the mEST and ZET.

For the AhR CALUX assay, the results of luminescence measurement were expressed as relative light units (RLUs). All data are expressed as induction factor by dividing the mean RLUs of exposed wells to the mean RLUs of the corresponding solvent control (0.25% v/v DMSO). EC50 (effective concentration inducing 50% response) values were calculated using Graphpad from the concentration–response curves of the AhR CALUX assay. For the mEST, the results of cell viability were expressed as the percentage of cell viability as compared to the solvent control which was set as 100%. Data from the ES-D3 cell differentiation assay were expressed as the percentage of fraction of total differentiated EBs into contracting cardiomyocytes, out of the total EBs plated in the 24-well plate. For the ZET, results were expressed as a percentage response of the total extended-GMS score (maximum 23) and as embryo survival at 96 hpf.

To determine the benchmark concentrations (BMC), concentration–response curves obtained in the mEST and ZET were fitted to all quantal concentration–response models from the European Food Safety Authority (EFSA) benchmark dose (BMD) modelling web-tool (https://shiny-efsa.openanalytics.eu/), based on the R-Package PROAST version 66.40 developed by the Dutch National Institute for Public Health and the Environment (RIVM). The quantal models of the ESFA BMD modelling web tool include two-stage, log-logistic, weibull, log probit, gamma, logistic, probit, exponential, and hill models. The benchmark response (BMR) was set to 50% (i.e., BMC50), representing the concentration that induced 50% inhibition of EBs differentiated into contracting cardiomyocytes in the mEST, 50% reduction of the embryo survival in the ZET, or 50% reduction in the extended-GMS score in the ZET. The performance of each fitted model was evaluated based on the goodness-of-fit, the scaled residuals, and the visual inspection of model fitting. The final BMC50 values were selected from the accepted model with the lowest Akaike’s Information Criterion (AIC) (Haber et al. 2018) (Supplementary material 3).

### Linear regression analysis

Correlation between the AhR-mediated activities (EC50) and in vitro developmental toxicity potencies in mEST or ZET (BMC50) of the DMSO extracts of the petroleum substances was made using linear regression analysis in GraphPad Prism 5.0. Data obtained in the present study were combined with data obtained from previous studies (Kamelia et al. 2017, 2018, 2019a, 2019b), which tested a range of petroleum substances extracts (within and across product categories) in the AhR CALUX assay, mEST and ZET. No EC50 or BMC50 could be derived for petroleum substances that induced no substantial effects in the AhR CALUX assay, mEST and ZET, and therefore these samples were not included in the correlation test. The obtained R^2^ reflects the goodness-of-fit of data to the fitted regression, and the compared data were considered significantly different if the p value was lower than 0.05.

## Results

### Effects of the DMSO extracts of sixteen petroleum substances in the AhR CALUX assay

**Fig. 2:**
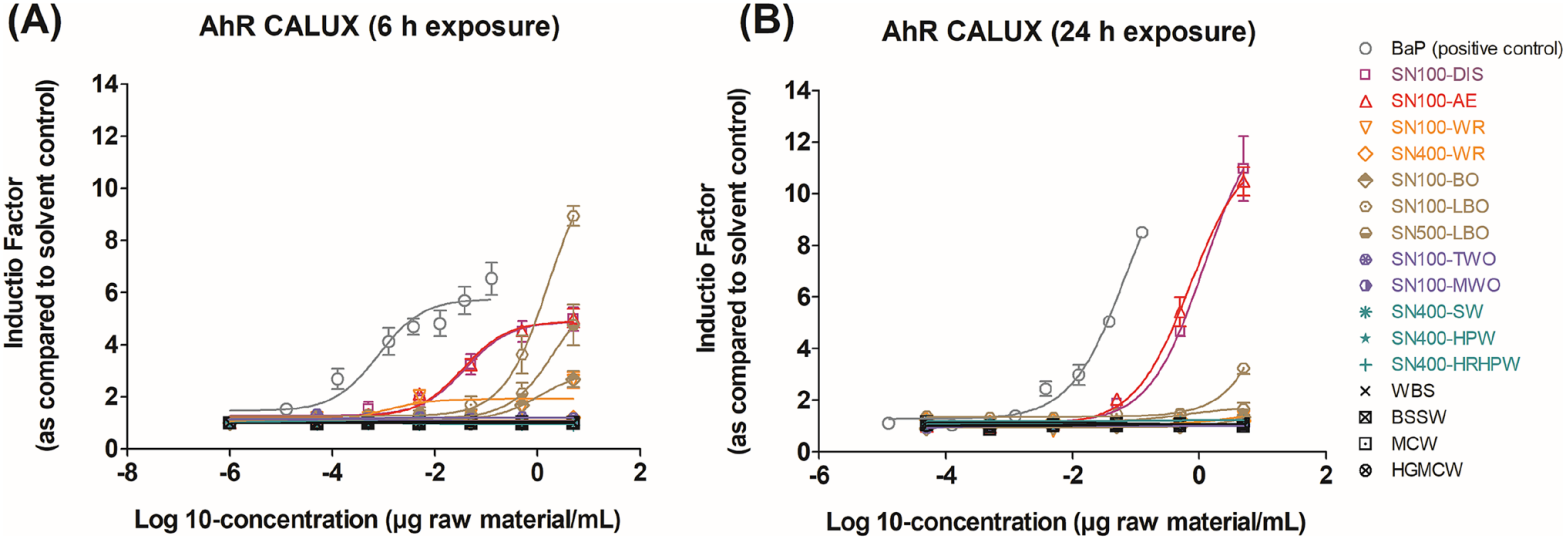
Concentration-dependent effects of BaP (positive control) and the DMSO extracts of sixteen petroleum substances in the AhR CALUX assay following **A** 6 h and **B** 24 h exposure. Results represent data from three independent experiments and are presented as mean ± SEM

The AhR-mediated activities of the DMSO extracts of sixteen petroleum substances were evaluated in the AhR CALUX assay with two exposure time windows, 6 h and 24 h. The concentration–response curves of the AhR-mediated activities of all test samples are shown in Fig. 2. Results show that samples SN100-DIS and SN100-AE (with EC50-6 h of 0.053 μg/mL and 0.050 μg/ml, respectively; with EC50-24 h of 0.25 μg/ml and 0.19 μg/ml, respectively) were the most potent test substances under study to induce AhR-mediated activity in the AhR CALUX assay following 6 h and 24 h exposure. Two samples, namely SN100-LBO and SN500-LBO, induced AhR-mediated activities following 6 h exposure (see their EC50s in Table 1), but such activities were not observed after 24 h exposure, indicating a transient AhR activation in the AhR CALUX assay. The other twelve samples tested negative in the AhR CALUX assay after 6 h and 24 h exposure.

**Table 1:** Overview of the EC50 and BMC50 values of the DMSO extracts of sixteen petroleum substances under study in the AhR CALUX assay, mEST and ZET

Test compound	EC50 of AhR CALUX agonist assay (6 h exposure, μg/ml)	EC50 of AhR CALUX agonist assay (24 h exposure, μg/ml)	BMC50 of the mEST (µg/ml)	BMC50 of the ZET (µg/ml)	
				Embryo lethality	Extended-GMS
SN100-DIS	0.053	0.25	15.3	18.1	9.72
SN100-AE	0.050	0.19	3.21	7.41	3.91
SN100-WR	–	–	–	–	–
SN400-WR	–	–	–	–	–
SN100-BO	–	–	–	–	–
SN100-LBO	0.21	–	> 250 μg/ml ^a^	> 250 μg/ml ^a^	–
SN500-LBO	1.17	–	> 250 μg/ml ^a^	–	–
SN100-TWO	–	–	–	–	–
SN100-MWO	–	–	–	–	–
SN400-SW	–	–	–	–	–
SN400-HPW	–	–	–	–	–
SN400-HPHPW	–	–	–	–	–
WBS	–	–	–	–	–
BSSW	–	–	–	–	–
MCW	–	–	–	–	–
HGMCW	–	–	–	–	–

Note: “-” means the sample was tested negative in the AhR CALUX assay, mEST and ZET

^a^The calculated BMC50s were higher than the highest tested concentration of 250 μg/ml

### Effects of the DMSO extracts of sixteen petroleum substances in the mEST

**Fig. 3:**
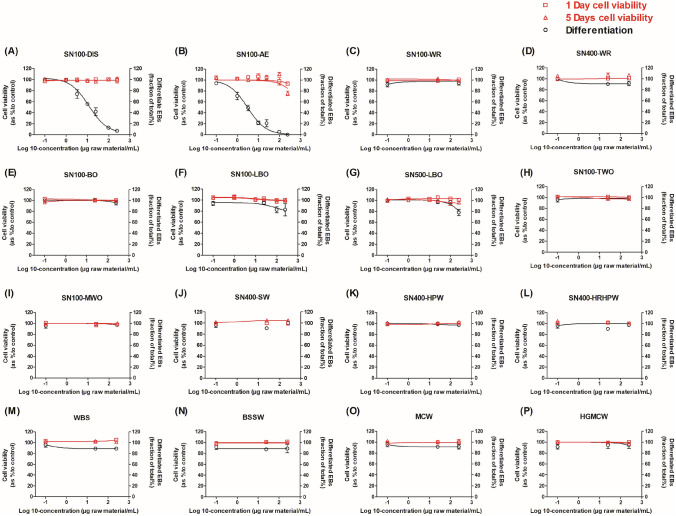
Concentration-dependent effects of the DMSO extracts of sixteen petroleum substances on ES-D3 cell viability upon 1 day (red line with unfilled square symbols) and 5 days (red line with unfilled triangle symbols) exposure, and on inhibition of ES-D3 cell differentiation into contracting cardiomyocytes (black line with round symbols). Results represent data from at least three independent experiments and are presented as mean ± SEM

The in vitro developmental toxicity of the test samples was first evaluated in the mEST. As illustrated in Fig. 3, none of the test samples induced cytotoxicity to the ES-D3 cells following 1- or 5-day exposure, up to the highest concentration tested (250 μg/ml). Only two test samples: SN100-DIS (Fig. 3A) and SN100-AE (Fig. 3B) inhibited the differentiation of the ES-D3 cells into contracting cardiomyocytes. The BMC50 values derived from the concentration–response curves of SN100-DIS and SN100-AE in the ES-D3 cell differentiation assay were 15.3 μg/ml and 3.21 μg/ml, respectively (Table 1). Samples SN100-LBO and SN500-LBO inhibited the ES-D3 cell differentiation with ~ 20% reduction at the highest tested concentration of 250 μg/ml, thus their BMC50s were above 250 μg/ml. The other samples tested negative in the ES-D3 cell differentiation assay of the mEST.

### Effects of DMSO extracts of the sixteen petroleum substances in the ZET

**Fig. 4:**
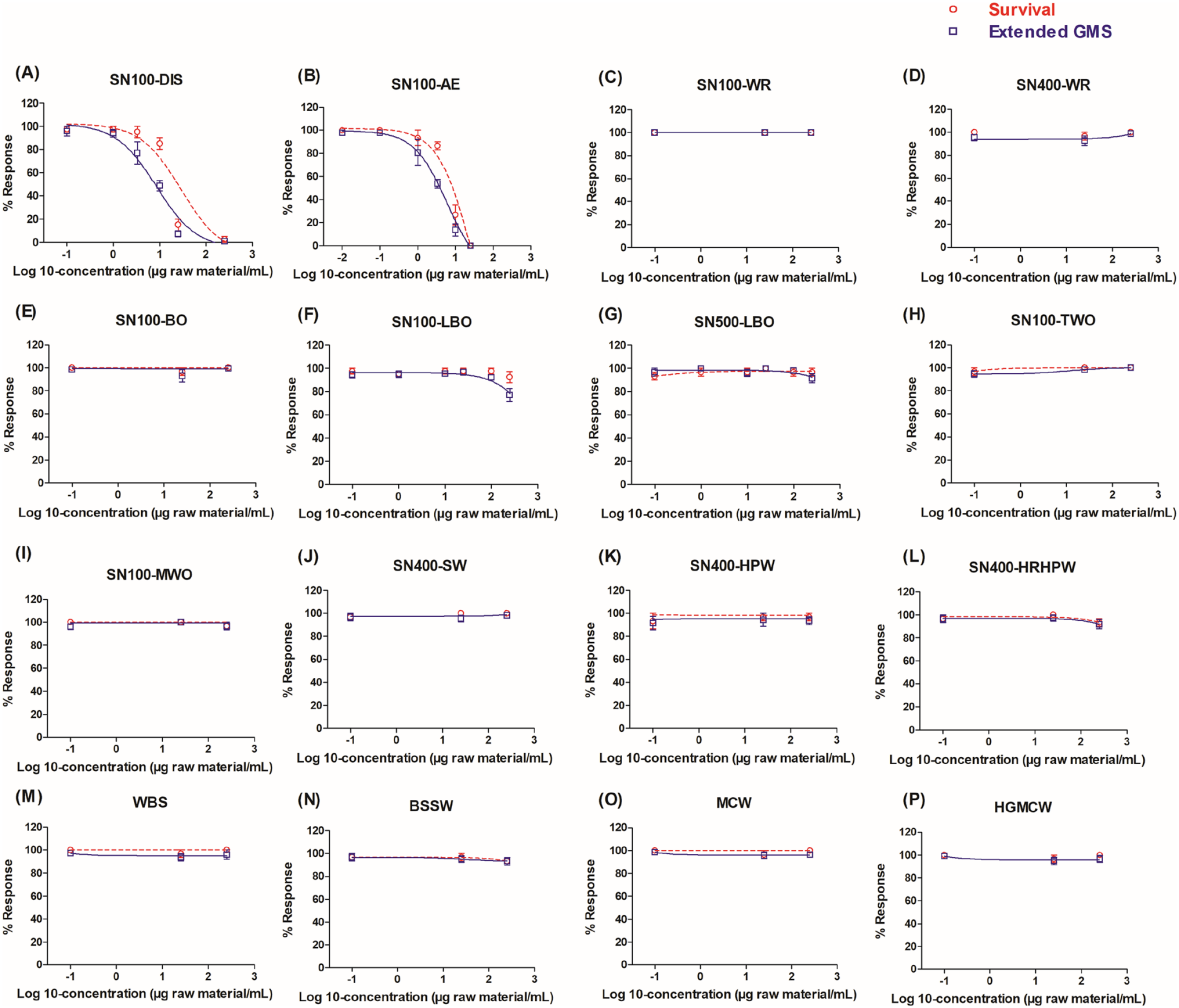
Concentration-dependent effects of the DMSO extracts of the sixteen petroleum substances on zebrafish embryo survival (red line with unfilled round symbols) and extended-GMS (blue line with unfilled square symbols). Results represent data from at least three independent experiments and are presented as mean ± SEM

**Fig. 5:**
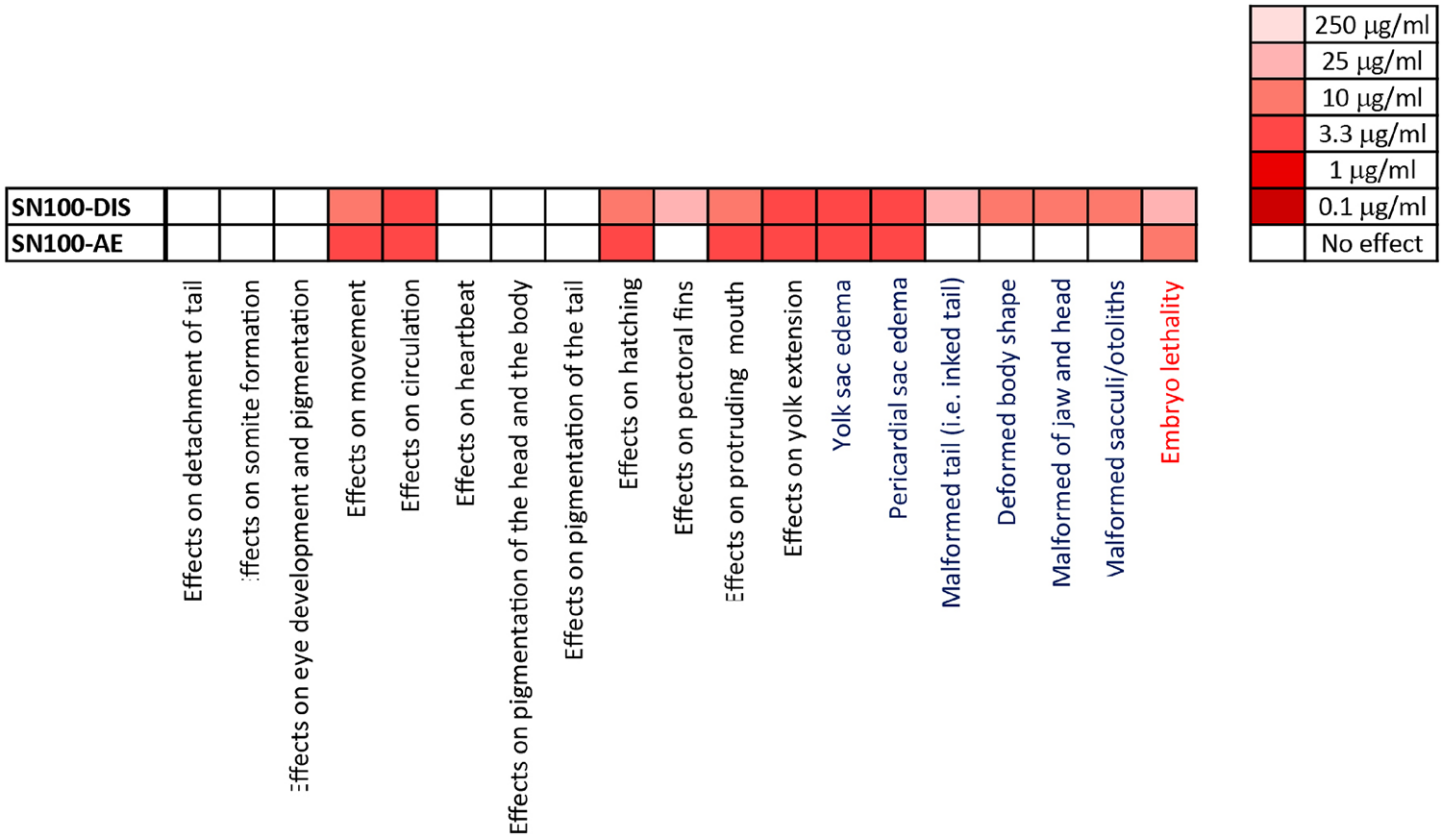
Heatmap summarizing the results in the ZET for the 18 endpoints scored based on the general morphology development (black font), dysmorphogenic (blue font) and for embryo lethality (red font) endpoints, upon exposure to the DMSO extracts of sample SN100-DIS and SN100-AE at 96 hpf. An exposure was considered as a ‘hit’ for a specific endpoint on the heatmap when ≥ 50% of the viable embryos showed effects on this endpoint scored at 96 hpf. The lowest concentration of the respective test compound that corresponds to a ‘hit’ is indicated by the color code shown on the right part of the figure. Results presented in the heatmap represent data from at least three independent experiments

The ZET was also applied to investigate the in vitro developmental toxicity of the DMSO extracts of sixteen petroleum substances under study. Figure 4 shows the concentration-dependent effects of the DMSO extracts of sixteen petroleum substances in the ZET based on embryo survival and the extended GMS at 96 hpf. The results show that only two test compounds: SN100-DIS and SN100-AE induced substantial reduction in both the embryo survival and the extended-GMS responses at 96 hpf. The BMC50 values for embryo survival and extended-GMS, derived from the concentration–response curves of SN100-DIS and SN100-AE in the ZET, are listed in Table 1. The calculated BMC50 value of SN100-DIS for embryo survival was 18.1 μg/ml and that for the extended GMS was 9.72 μg/ml. The BMC50 value of SN100-AE for embryo survival was 7.41 μg/ml and that for the extended GMS was 3.91 μg/ml. Sample SN100-LBO induced ~ 20% reduction in the extended-GMS response just at the highest tested concentration of 250 μg/ml, thus its BMC50 was above 250 μg/ml. The other thirteen samples tested negative in the ZET. Furthermore, a heatmap (Fig. 5) was generated to better visualize the affected endpoints induced by samples SN100-DIS and SN100-AE in the ZET. As depicted in Fig. 5, the most affected endpoints upon exposure to SN100-DIS and SN100-AE include the absence of movement and circulation, unhatched embryos, yolk extension was not fully emptied, pericardial sac and yolk sac edema, and cumulative mortality.

### *Correlation between *in vitro* potencies obtained in the AhR CALUX assay and/or mEST and ZET*

To investigate the potential relationship between the AhR-mediated activities, quantified in the AhR CALUX assay, and the in vitro developmental toxicity of the DMSO extracts of the petroleum substances in the mEST or ZET, a correlation analysis was performed by combining the AhR CALUX assay, mEST and ZET results obtained in the present study with results obtained in a series of previous studies (Kamelia et al. 2017, 2018, 2019a, 2019b) which tested different categories of petroleum substances samples including straight run gas oil (GO), vacuum tower overhead or vacuum gas oil (VTO), distillate aromatic extract (DAE), residual aromatic extract (RAE), and heavy fuel oil (HFO) in the same in vitro assays. It should be noted that since the previous studies only tested the AhR-mediated activities of petroleum substances extracts following 6 h exposure, the linear regression analysis was only performed between the in vitro potencies in mEST or ZET and AhR-mediated activities following 6 h exposure. Moreover, in the previous studies when testing other categories of petroleum substances in the ZET (Kamelia et al. 2019a), the BMC50 was determined based on GMS instead of extended GMS. Therefore, to be consistent with this previous study, BMC50s based on GMS were also calculated for the samples that tested positive in the ZET in the present study (i.e., poorly refined SN100-DIS and SN100-AE). Their BMC50s based on the GMS are listed in Table 1, and the concentration–response curves on embryo survival, GMS and extended GMS of these two samples are presented in Supplementary material 2.

**Fig. 6:**
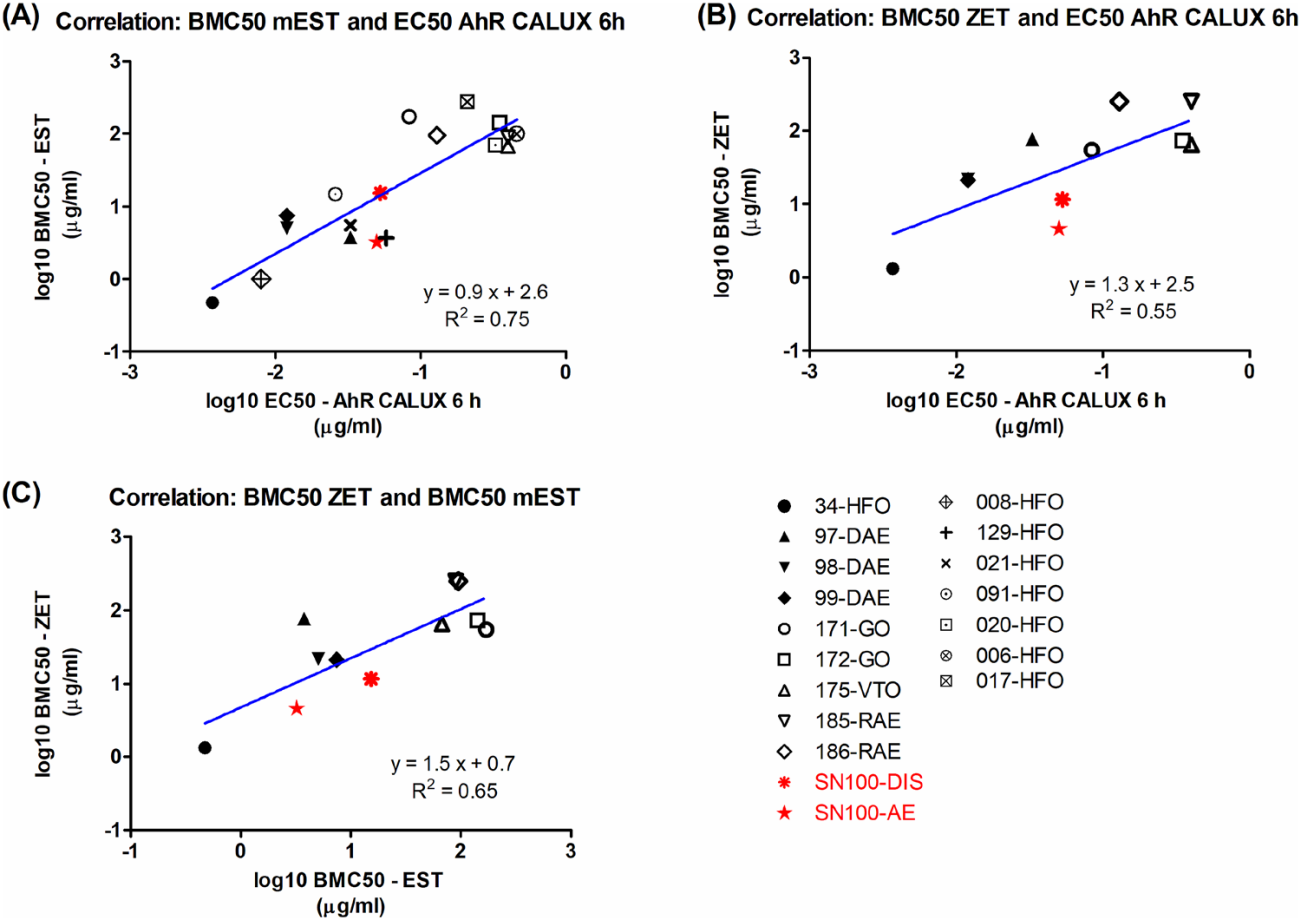
Correlation between the AhR mediated activities following 6 h exposure (expressed as EC50s) of the DMSO extracts of petroleum substances as tested in the AhR CALUX assay and their in vitro developmental toxicity potency as tested in the **A** mEST (expressed as BMC50s) and **B** ZET (expressed as BMC50s); and **C** correlation between the in vitro developmental toxicity potency in the mEST and ZET. Note: the in vitro data of samples 34-HFO, 97-DAE, 98-DAE, 99-DAE, 171-GO, 172-GO, 175-VTO, 185-RAE, 186-RAE, 008-HFO, 129-HFO, 021-HFO, 091-HFO, 020-HFO, 006-HFO and 017-HFO were taken from Kamelia et al. 2017, 2018, 2019a, 2019b

The results (Fig. 6A and B show that when the data of the present study are combined with those from previous studies by Kamelia et al. (2017, 2018, 2019a), the current correlation between the AhR-mediated activities (following 6 h exposure) and the in vitro developmental toxicity potencies in the mEST (R^2^ = 0.75; Fig. 6A) or ZET (R^2^ = 0.55; Fig. 6B) matches the correlation defined previously (mEST and AhR CALUX assay: *R*^2^ = 0.80; ZET and AhR CALUX assay: *R*^2^ = 0.66) (Kamelia et al. 2017, 2018, 2019a). Moreover, Fig. 6C shows that the correlation between the combined mEST and ZET results (*R*^2^ = 0.65) also matches the correlation defined previously (Kamelia et al. 2019a).

## Discussion

Previous studies have shown the usefulness of a battery of in vitro assays to evaluate the developmental toxicity of PAC-containing substances, including highly complex petroleum substances extracts (Kamelia et al. 2017, 2018, 2019a, 2019b, 2021). The present study aimed to extend the usefulness of such a battery of in vitro assays including the AhR CALUX assay, mEST and ZET for developmental toxicity testing of a series of petroleum substance extracts from different product categories and varying in their PAC content. To this end, DMSO extracts of sixteen test compounds, including two poorly refined petroleum substances, seven highly refined petroleum substances and seven petroleum-derived waxes were tested. Results obtained show that the two poorly refined petroleum substances i.e., SN100-DIS and SN100-AE, induced concentration-dependent in vitro developmental toxicity in both mEST and ZET, and sustained AhR activation in the AhR CALUX assay. Two base oil extracts (i.e., SN100-LBO and SN500-LBO) induced only transient AhR activation following 6 h exposure since such activity was not seen upon 24 h exposure in the AhR CALUX assay. The remaining petroleum substances and petroleum-derived waxes tested, containing either very low levels or no PACs at all, tested negative in all assays applied (AhR CALUX assay, mEST and ZET). Due to the absence of in vivo developmental toxicity data on the actual samples, it is not possible to directly assess the correlation between in vitro developmental toxicity potency, obtained in mEST and ZET, and potencies observed in vivo. Nonetheless, our results demonstrate that the applied test battery does not only capture the in vitro developmental toxicity induced by DMSO extracts of PAC-containing petroleum substances, but also the absence of developmental toxicity-related effects for substances containing virtually no aromatics and no PACs, such as highly refined oils and petroleum-derived waxes.

Sample SN100-DIS is a vacuum distillate, produced by the vacuum distillation of the residuum from atmospheric distillation of crude oil, and belongs to the petroleum substance category of untreated/acid-treated oils (UATO). Vacuum distillates that have not been subject to further refining processes may thus contain hazardous 3- to 7-ring PACs, typically ≤ 7% wt % (HPV 2012; Dalbey et al. 2014; Table S4). Subsequent refining processes of the vacuum distillate, such as solvent extraction, results in two streams: aromatic extract substances (sample SN100-AE) containing typically high levels of total 3- to 7-ring PACs, typically ≤ 20% (Dalbey et al. 2014; HPV 2014; Table S4) and waxy raffinate substances (samples SN100/400-WR) which serves as the primary feedstock for producing base oils and wax substances (HPV 2012; Carrillo et al. 2021, 2022). Waxy raffinate substances typically contain low level of aromatics including PACs (typ. ≤ 0.5%, see Fig. 1 and Table S4) since they are sufficiently refined (Dalbey et al. 2014). In line with the fact that the samples SN100-DIS and SN100-AE were not subject to further refining and are expected to contain substantial amounts of 3- to 7-ring PACs, both samples were able to induce in vitro developmental toxicity in the ZET and mEST. The waxy raffinate samples tested (samples SN100-WR and SN400-WR), which contain low levels of PACs, were negative in all assays applied in the present study. The positive outcomes for SN100-DIS and SN100-AE are in line with in vivo studies which reported that the developmental toxicity induced by some petroleum substances is associated with their 3- to 7-ring PAC content (Feuston et al. 1994; Murray et al. 2013). In addition, the correlation observed between the AhR-mediated activity (following 6 h exposure) and the in vitro developmental toxicity in the ZET (*R*^2^ = 0.55) or mEST (*R*^2^ = 0.75) when combining results of SN100-DIS and SN100-AE with petroleum substances from other categories matches with the correlation defined previously by Kamelia et al. 2017, 2018, 2019a. Altogether, the results support the hypothesis that the AhR plays an important role in mediating the developmental toxicity of petroleum substances containing relatively high levels of 3–7 ring PAC (Kamelia et al. 2018, 2019b). The EC50 values of SN100-DIS and SN100-AE obtained in the AhR CALUX assay following 24 h exposure were higher than the EC50 values following 6 h exposure, indicating that sustained AhR activation by these two substances remained even after prolonged (24 h) exposure. It is known that persistent AhR activation such as the activity induced by strong AhR ligand TCDD could lead to various adverse effects including developmental toxicity, while transient AhR induction is consistent with maintaining normal cell homeostasis (Hankinson 1995; Puga et al. 2002; Mitchell and Elferink 2009; Bock 2013; Larigot et al. 2018). However, a previous study by Kamelia et al. 2018 only measured the AhR-mediated activity after 6 h but not 24 h exposure, hence comparison and correlation between the in vitro developmental toxicity of the petroleum substances and their sustained AhR activation cannot be further evaluated. Therefore, it seems of interests to include the AhR CALUX assay with two exposure time windows (i.e., 6 h and 24 h) in the proposed in vitro testing strategy for developmental toxicity testing of highly complex petroleum substances.

Dewaxing of waxy raffinates separates the waxes from the oils, thus yielding two streams namely slack wax (sample SN400-SW) and base oil (samples SN100-BO, SN100-LBO and SN500-LBO). Base oil substances may contain up to 1.4% total PACs (Fig. 1, Table S4). The major hydrocarbon components of slack wax are paraffins and low levels of highly alkylated 1- to 2-ring aromatics (CONCAWE 2017; Carrillo et al. 2019). Slack wax could be de-oiled into hard paraffin wax substances (sample SN400-HPW), and then generate highly refined hard paraffin wax substances (sample SN400-HRHPW) by hydrotreatment. It was shown that waxes-related products (e.g., slack wax, paraffin wax) generated from sufficiently refined lubricating oil base stocks contain no or very low PAC (up to 0.2%, Table S4)(HPV 2011, 2014b; Dalbey et al. 2014). All these petroleum substances-derived waxes (i.e., SN400-SW, SN400-HPW, SN400-HRHPW) tested negative in the AhR CALUX assay, mEST and ZET. No in vivo developmental toxicity study have been done previously with petroleum-derived wax materials, but in principle, the developmental toxicity potency of these substances is expected to be negligible if they are sufficiently refined (HPV 2011; Dalbey et al. 2014). The saturated hydrocarbons present in wax materials are related to those in base oils because they are derived from the same vacuum distillate streams, thus their potential developmental toxicity can be assessed using read-across from data with base oils (HPV 2011). Developmental toxicity studies conducted with base oils, either via oral (up to 2000 mg/kg/day; Mobil, 1987b) or dermal (at 1000 mg/kg/day; Kuhl et al. 2010) administration, show no developmental-related effects in the offspring of pregnant rats. In agreement with that observation, the DMSO extracts of the base oil samples tested (samples SN100-BO, SN100-LBO and SN500-LBO) induced no developmental toxicity in the ZET or mEST of the present study.

It is interesting to observe that two of the three base oil samples, SN100-LBO and SN500-LBO, induced a transient AhR activation in the AhR CALUX assay following 6 h exposure. The predominant class of aromatics in base oils is formed by non-hazardous highly alkylated 1- or 2-ring aromatics (Carrillo et al. 2022). Thus, the transient AhR activation induced by lubricating base oil could be explained by the presence of this type of highly alkylated 1- or 2-ring aromatics in these substances or most likely by the residual levels of 3–7 PAC still present in these oils but not high enough to sustain this effect. HRBO are produced by hydrotreating the base oils, either once (SN100-TWO) or twice (SN100-MWO), respectively. Both SN100-TWO and SN100-MWO tested negative in the AhR CALUX assay, mEST, and ZET in the present study, which is in line with previous studies that reported that HRBO tested negative for developmental toxicity both in vivo and in vitro (Mobil 1987b; Feuston et al. 1994; Kamelia et al. 2020). Waxy brightstock, a heavy lubricating stock, is derived from vacuum residuum (Wauquier 1995). Waxy bright stock substances could be further processed into bright stock slack wax and microcrystalline wax by dewaxing, deoiling and/or hydrotreatment. All these petroleum substances-derived waxes (samples WBS, BSSW, MCW, HGMCW) tested negative in the AhR CALUX assay, mEST, and ZET. Altogether, our results indicate that refining processes efficiently reduced the amounts of hazardous PACs (mainly 3- to 7-ring PACs) in waxy raffinates and the subsequent products down to a level that induced no developmental toxicity even if 1- or 2-ring aromatics are present.

In the AhR CALUX assay, exposure time windows of 6 and 24 h were applied to assess the AhR-mediated activities of the DMSO extracts of sixteen petroleum substances. Previous studies demonstrated that 6 h is a standard/sufficient exposure time for the detection of AhR induction by benzo[a]pyrene-like compounds (Vrabie et al. 2009) and also PAH-containing petroleum extracts (Machala et al. 2001; Kamelia et al. 2018, 2019b, 2021) in the AhR CALUX assay, whereas 24 h exposure should be applied for more persistent compounds (e.g., TCDD) that require a prolonged time period to induce AhR activation (Vrabie et al. 2009). The DMSO extracts of the sixteen petroleum substances under study are PAC-containing materials, and therefore 6 h was included as one of the exposure time windows in the present AhR CALUX assay. The highest AhR induction by benzo[a]pyrene, the positive control of the AhR CALUX assay, and DMSO-extracts of petroleum substances under study (at the highest tested concentration of 5 μg raw material/ml), was obtained after 6 h of exposure (Fig. 2). Furthermore, by using both 6 and 24 h exposure time windows, the present study also investigated whether the observed AhR induction is of transient or sustained nature. This is relevant because transient AhR activation is considered to be a protective adaptive response, whereas persistent AhR activation may affect normal biological processes including embryonic development in vertebrates (Mitchell and Elferink 2009; Bock 2013). For samples SN100-DIS and SN100-AE in the AhR CALUX assay, AhR induction was observed after 6 h exposure, and this response remained after 24 h (or prolonged) exposure time, indicating a sustained AhR activation by both substances. However, the EC50 values tested at 6 h exposure were lower than those at 24 h exposure (see Table 1). This is mainly due to the presence of cytochrome P450 (CYP) 1A activity in the H4IIE.luc cells used for the AhR CALUX assay. This leads to the degradation of easily metabolizable compounds, including PAHs, over time, and consequently results in decreased AhR activation after 24 h (or prolonged) exposure time (Vrabie et al. 2009). As a result, the EC50 values obtained following 24 h exposure to BaP and PAH-containing materials in the AhR CALUX assay are higher than those upon 6 h exposure (EC50 BaP_6h: 6 × 10^–4^ μg/ml; EC50 BaP_24h: 1.8 × 10^–2^ μg/ml). In line with this observation, it was previously reported that BaP induction is higher after 6 h than after 24 h in the AhR CALUX assay, whereas the reverse was observed for more persistent compounds like TCDD (Hamers et al. 2000; Machala et al. 2001; Vrabie et al. 2009; Fang et al. 2023). On the contrary, for base oil samples tested (SN100-LBO, SN500-LBO, SN100-BO), the AhR activity was observed only after 6 h but not 24 h exposure, implying a transient or short-lived AhR activation.

It should be mentioned that the refined petroleum substances generated from the same crude oil feedstock could have a different viscosity, for example SN100 products have lower viscosity compared to SN400/500 products. Petroleum substances with higher viscosity may contain predominantly high molecular weight substituted PACs (e.g., with long alkyl side chains) because high molecular weight is related to alkyl chain length (Carrillo et al. 2022). On the other hand, the hazardous aromatics are typically the 3- to 7-ring PACs with no or low degree of alkylation, that can be efficiently and selectively removed from feedstock during the refining processes (e.g., solvent extraction and hydrotreatment). However, the highly alkylated 1- or 2-ring aromatics are not removed completely and may remain in the raffinate. They are intrinsic constituents of some petroleum substances, particularly those with high viscosity but do not pose developmental toxicity-related concerns (Carrillo et al. 2022). To counter the influence of viscosity and alkylation in experimental set ups, DMSO serves as an ideal tool to selectively extract those PAC with potential biological activity from those aromatics which tend to remain in the raffinate. Therefore, the DMSO extracts of petroleum substances, instead of neat/bulk materials, were used to introduce the test materials into the in vitro test systems applied in the present study. DMSO selectively extracts and concentrates the PACs present in petroleum substances, which consist of mainly 3- to 7-ring PACs that are either unsubstituted (naked) or contain short-chain alkyl substituents (Roy et al. 1988; Carrillo et al. 2019). This 3- to 7-ring PAC fraction was shown to be the main group of constituents responsible for the observed developmental toxicity of poorly refined petroleum substances (Kamelia et al. 2017). The non-extracted fraction from the DMSO extraction procedure, known as the raffinate fraction, contains saturated hydrocarbons and lower aromatics (i.e., 1- to 2-ring) that are highly alkylated (Mackerer et al. 2003; Carrillo et al. 2019). The use of DMSO extracts for in vitro developmental toxicity testing of petroleum substances has been applied previously and a good correlation exists between obtained in vitro results for the DMSO extracts and available in vivo results for the parent compounds (Kamelia et al. 2017, 2018, 2019a, 2019b, 2021). Furthermore, a comparison of the PAC profiles between the undiluted petroleum substances and the corresponding DMSO extracts indicates substantial similarities (Luo et al. 2020). Altogether, the use of DMSO-extracts for in vitro dosing of petroleum substances proved to adequately capture the biologically relevant fraction and thus the expected hazards, including developmental toxicity, of these substances (CONCAWE 1992; Dalbey et al. 2014; Carrillo et al. 2019; House et al. 2021).

Most petroleum substances are produced or registered at a volume of ≥ 1000 tonnes/year. As a consequence, they are required to be tested for their effects on prenatal development under the EU REACH (Registration, Evaluation, Authorization, and Restriction of Chemicals) regulation. If this is all done according to the current OECD TG 414 (OECD 2018), a huge number of experimental animals (rodent and non-rodent) and resources would be required. Currently, there is no standardized testing method established for assessing the developmental toxicity of highly complex materials like petroleum substances, as it is still unclear what lowest level of total 3- to 7-ring PACs in the petroleum substances would induce in vivo developmental toxicity. It is worth noting that the samples tested negative for in vitro developmental toxicity in this study are all raffinates or derived from raffinates (the primary feedstock for producing base oils and wax substances), and all contains relatively low level or no PACs (Table S4). Regarding the toxicity of PACs, results obtained in the present study further demonstrate that the group of 3- to 7-ring PACs (naked and/or lowly alkylated), is not only associated with mutagenicity/carcinogenicity of poorly refined petroleum substances (Roy et al. 1988; McKee et al. 1989; Carrillo et al. 2019), but also relevant for their developmental toxicity. Therefore, it can be assumed that the relevant analytical methods, such as PAC/H (aromatic ring class/ARC) and/or the IP346 test, could be used and extended to screen the developmental toxicity of petroleum substances as well. Altogether, the proposed in vitro testing battery (i.e., AhR CALUX assay, mEST and ZET) in combination with the simple yet robust analytical methods may be applied to evaluate the developmental toxicity potency of petroleum substances. Such a joint approach might provide a more reliable prediction than using only the battery of in vitro assays, and help with screening the in vitro developmental toxicity of petroleum substances from different categories to set priorities for subsequent in vivo testing. The applicability of such a joint approach, especially the cut-off for total PAC (%) and in vitro developmental toxicity potency obtained from the aforementioned battery, would need to be further investigated.

In conclusion, the present study extends the usefulness of a test battery, including the AhR CALUX assay, mEST and ZET for the developmental toxicity testing (and mode-of-action investigation) of extracts of highly complex petroleum substances, produced by different refining processes. Our results suggest that the highly refined petroleum products with extremely low level or devoid of PACs do not induce developmental toxicity and confirm that the persistent activation of the AhR is relevant to the in vitro developmental toxicity of the unrefined petroleum substances obtained in the mEST and ZET. Data obtained from the proposed in vitro testing strategy together with the IP346 test could be further used to facilitate read-across from petroleum substances for which in vivo data are already available, to the petroleum substances for which in vivo data are lacking, thereby ultimately contributing to reducing the number of experimental animals required to assess developmental toxicity of petroleum substances regulated under REACH.

### Supplementary Information

Below is the link to the electronic supplementary material.

Supplementary file1 (DOCX 1143 KB)

## Data Availability

All data of this study are available within the paper and its Supplementary Materials. Raw data are available from the corresponding author upon requests.
