# Supplementary material for: Evaluating the in vitro developmental toxicity potency of a series of petroleum substance extracts using new approach methodologies (NAMs)
Source: Arch Toxicol. 2023 Dec 12;98(2):551–65. doi: 10.1007/s00204-023-03645-7 (PMC10794406; doi:10.1007/s00204-023-03645-7)
Supplement: Supplementary file 1 — Supplementary file1 (DOCX 1143 KB) [file 204_2023_3645_MOESM1_ESM.docx]

**Evaluating the in vitro developmental toxicity potency of a series of petroleum substance extracts using new approach methodologies (NAMs)**

Jing Fang^1*^, Ivonne M.C.M. Rietjens^1^, Juan-Carlos Carrillo^2^, Peter J. Boogaard^1^, Lenny Kamelia^2^

^1^Division of Toxicology, Wageningen University and Research, 6708 WE, Wageningen, The Netherlands

^2^Shell Global Solutions International B.V., 2596 HR, The Hague, The Netherlands

*Corresponding author: Jing Fang ([jing.fang@wur.nl](mailto:jing.fang@wur.nl),  [ORCID](https://orcid.org/): 0000-0001-9213-5305)

**Supplementary material 1**

**Table S1**. The extended general morphological scoring (extended-GMS) system as described by Beekhuijzen et al. (2015). This scoring system contains two parts: 1) general development of zebrafish embryos (GMS) and 2) dysmorphogenic endpoints. In the general development part, endpoints no. 1-5 are evaluated at 24 hpf, endpoints 1-8 are evaluated at 48 hpf, endpoints 1-11 are evaluated at 72 hpf, and endpoints 1-12 are evaluated at 96 hpf. The 6 dysmorphogenic endpoints are only evaluated at 96 hpf.

| **No.** | **1). General Development Endpoints** | **Scores** | | | |
| --- | --- | --- | --- | --- | --- |
|  |  | **24 hpf** | **48 hpf** | **72 hpf** | **96 hpf** |
| 1 | Detachment of the tail | 2 | 3 | 3 | 3 |
| 2 | Somite formation | 1 | 1 | 1 | 1 |
| 3 | Eye development & pigment | 2 | 3 | 3 | 3 |
| 4 | Movement | 1 | 1 | 1 | 1 |
| 5 | Circulation | 1 | 1 | 1 | 1 |
| 6 | Heartbeat |  | 1 | 1 | 1 |
| 7 | Pigmentation of the head and the body |  | 1 | 1 | 1 |
| 8 | Pigmentation of the tail |  | 1 | 1 | 1 |
| 9 | Hatching |  |  | 1 | 1 |
| 10 | Pectoral fin |  |  | 1 | 1 |
| 11 | Protruding mouth |  |  | 2 | 2 |
| 12 | Yolk extension nearly empty |  |  |  | 1 |
|  | Total GMS score | 7 | 12 | 16 | 17 |
|  | | | | | |
| **No** | **2). Dysmorphogenic endpoints** | **Scores** | | | |
|  |  | **24 hpf** | **48 hpf** | **72 hpf** | **96 hpf** |
| 1 | No yolk sac edema |  | | | 1 |
| 2 | No pericardial edema |  |  |  | 1 |
| 3 | No malformed tail |  |  |  | 1 |
| 4 | No deformed body shape |  |  |  | 1 |
| 5 | No malformed head and jaw |  |  |  | 1 |
| 6 | No malformed sacculi/otoliths |  |  |  | 1 |


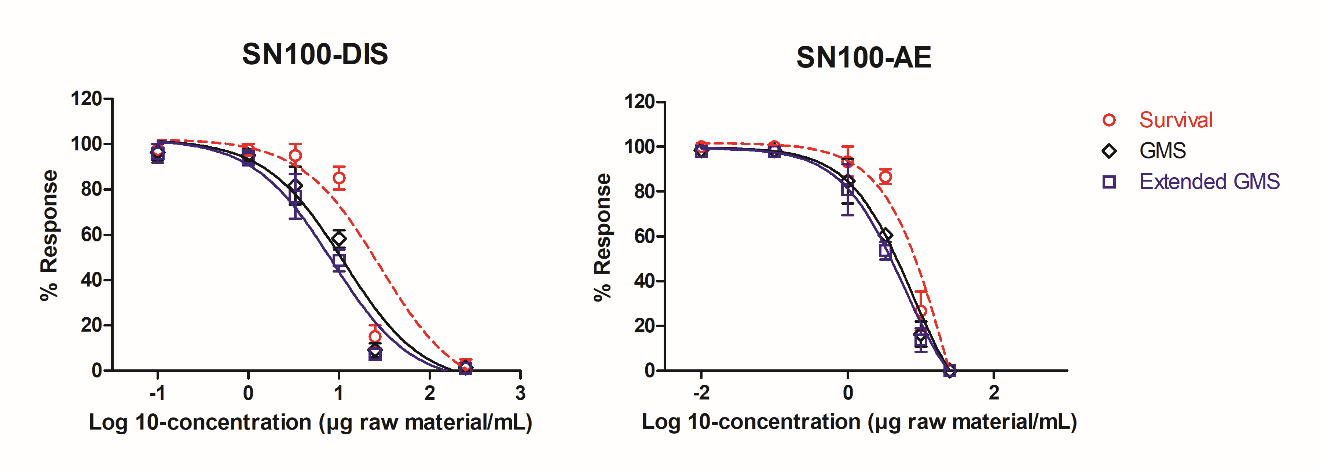
**Supplementary material 2**

BMC50_GMS_= 11.63 μg/ml μg/ml

BMC50_GMS_= 4.63 μg/ml

**Fig. S2.** Concentration-dependent effects of sample SN100-DIS and SN100-AE on embryo survival (red dotted line with unfilled circle symbol), GMS (black line with unfilled diamond symbols) and extended-GMS (blue line with unfilled square symbols) at 96 hpf in the ZET. Results represent data from 3 independent ZET experiments and are presented as mean percentage ± standard error of mean (SEM). BMC50_GMS_ values represent the concentration of SN100-DIS and SN100-AE that induced a 50% reduction in the GMS score in the ZET.

**Supplementary material 3**

Results from BMD analysis of the EST and ZET data for petroleum substances samples SN1—DIS and SN100-AE tested in the present study, based on the obtained EST (cell differentiation) and ZET (embryo lethality, GMS and extended-GMS at 96 hpf) data. Concentration-response curves obtained in the EST or ZET were fitted to all quantal concentration-response models (i.e., two-stage, log logistic, weibull, log probit, gamma, logistic, probit, exponential, and hill models) available in the EFSA BMD modelling web-tool (<https://shiny-efsa.openanalytics.eu/>). For BMC50 determination, the benchmark response (BMR) was set at 50%, representing the concentration that induces a 50% reduction of cell differentiation in the EST, and embryo lethality, GMS or extended GMS at 96 hpf in the ZET.

**Table. S2 (A)** Results from BMD analysis of the data on EST of SN100-DIS based on the EST cell differentiation data. The table presents characteristics of fitted models, the AIC values, and the benchmark concentration for 50% effect (BMC50). The model(s) with the lowest AIC value was selected.

| **model** | **No.par** | **loglik** | **AIC** | **accepted** | **BMDL** | **BMDU** | **BMD** | **conv** |
| --- | --- | --- | --- | --- | --- | --- | --- | --- |
| null | 2 | -128.68 | 261.36 |  | NA | NA | NA | NA |
| full | 9 | -66.56 | 151.12 |  | NA | NA | NA | NA |
| two.stage | 4 | -82.73 | 173.46 | no | NA | NA | 13.3 | yes |
| log.logist | 4 | -68.71 | 145.42 | yes | 9.64 | 24.0 | 15.1 | yes |
| Weibull | 4 | -70.33 | 148.66 | no | NA | NA | 20.1 | yes |
| **log.prob** | **4** | **-68.39** | **144.78** | **yes** | **9.79** | **24.1** | **15.3** | **yes** |
| gamma | 4 | -71.45 | 150.90 | no | NA | NA | 23.3 | yes |
| LVM: Expon. m3- | 4 | -70.97 | 149.94 | no | NA | NA | 20.0 | yes |
| LVM: Hill m3- | 4 | -70.95 | 149.90 | no | NA | NA | 20.0 | yes |


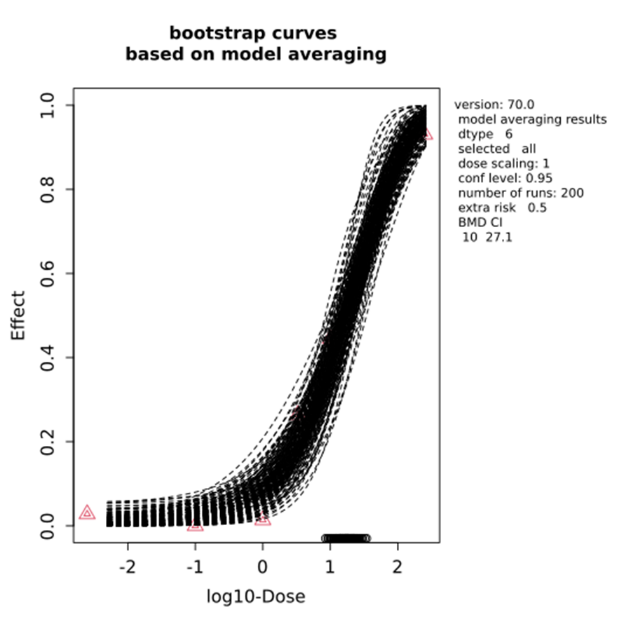

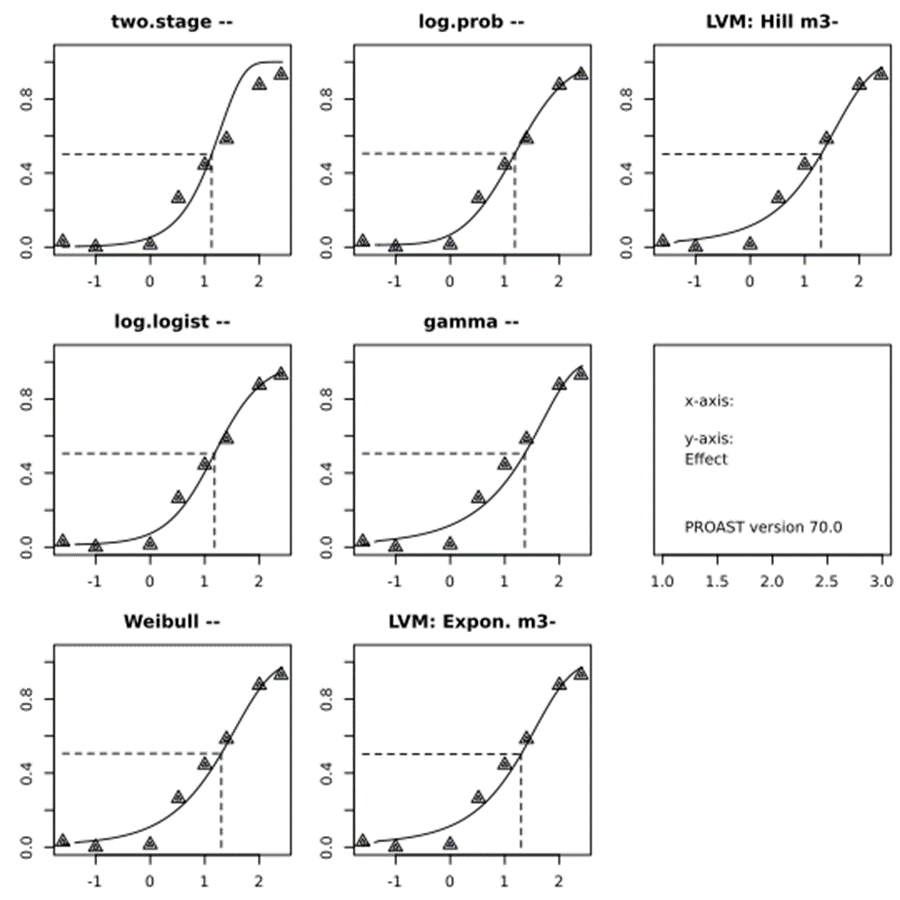


**Table. S2 (B)** Results from BMD analysis of the data on EST of SN100-AE based on the EST cell differentiation data. The table presents characteristics of fitted models, the AIC values, and the benchmark concentration for 50% effect (BMC50). The model(s) with the lowest AIC value was selected.

| **model** | **No.par** | **loglik** | **AIC** | **accepted** | **BMDL** | **BMDU** | **BMD** | **conv** |
| --- | --- | --- | --- | --- | --- | --- | --- | --- |
| null | 2 | -132.20 | 268.40 |  | NA | NA | NA | NA |
| full | 9 | -68.98 | 155.96 |  | NA | NA | NA | NA |
| two.stage | 4 | -81.04 | 170.08 | no | NA | NA | 10.30 | yes |
| log.logist | 4 | -70.05 | 148.10 | yes | 1.77 | 5.71 | 3.21 | yes |
| Weibull | 4 | -70.51 | 149.02 | yes | 2.03 | 7.07 | 3.93 | yes |
| **log.prob** | **4** | **-69.87** | **147.74** | **yes** | **1.76** | **5.82** | **3.21** | **yes** |
| gamma | 4 | -71.66 | 151.32 | no | NA | NA | 4.68 | yes |
| LVM: Expon. m3- | 4 | -70.83 | 149.66 | yes | 2.43 | 7.28 | 4.09 | yes |
| LVM: Hill m3- | 4 | -70.77 | 149.54 | yes | 2.00 | 7.22 | 3.92 | yes |


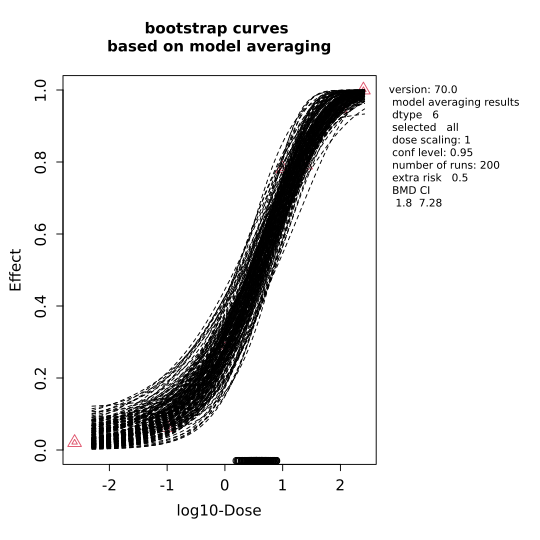

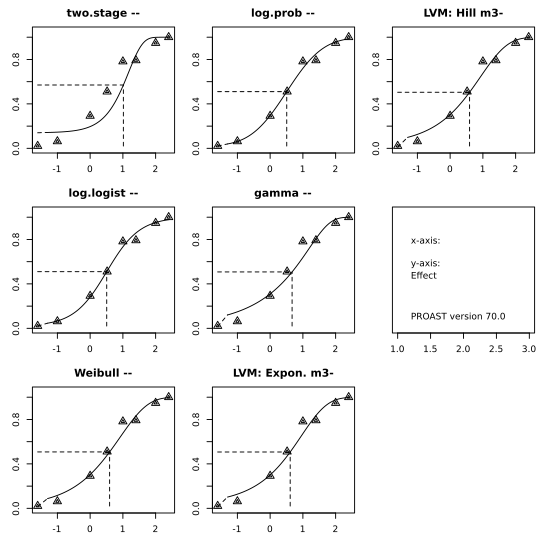


**Table. S2 (C)** Results from BMD analysis of the data on EST of SN100-DIS based on the embryo lethality data in the ZET. The table presents characteristics of fitted models, the AIC values, and the benchmark concentration for 50% effect (BMC50). The model(s) with the lowest AIC value was selected.

| **model** | **No.par** | **loglik** | **AIC** | **accepted** | **BMDL** | **BMDU** | **BMD** | **conv** |
| --- | --- | --- | --- | --- | --- | --- | --- | --- |
| null | 2 | -42.77 | 89.54 |  | NA | NA | NA | NA |
| full | 8 | -15.12 | 46.24 |  | NA | NA | NA | NA |
| two.stage | 4 | -16.03 | 40.06 | yes | 11.9 | 24.3 | 16.5 | no |
| log.logist | 4 | -16.24 | 40.48 | yes | 10.8 | 27.2 | 16.5 | yes |
| Weibull | 4 | -18.71 | 45.42 | no | NA | NA | 21.5 | yes |
| log.prob | 4 | -16.98 | 41.96 | no | NA | NA | 17.3 | yes |
| gamma | 4 | -18.76 | 45.52 | no | NA | NA | 21.6 | yes |
| **LVM: Expon. m3-** | **4** | **-15.69** | **39.38** | **yes** | **12.2** | **24.2** | **18.1** | **yes** |
| **LVM: Hill m3-** | **4** | **-15.69** | **39.38** | **yes** | **12.2** | **23.9** | **18.1** | **yes** |


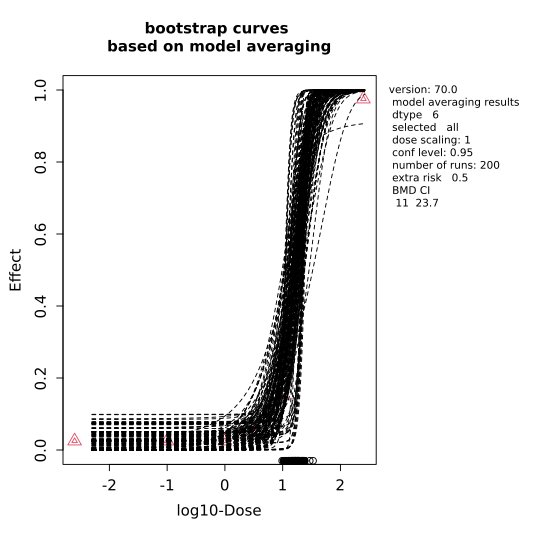

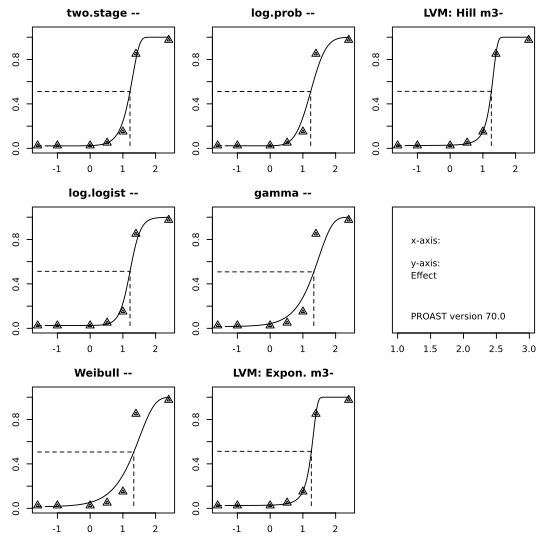
BMC50 = (18.1+18.1)/2=18.1

**Table. S2 (D)** Results from BMD analysis of the data on EST of SN100-AE based on the embryo lethality data in the ZET. The table presents characteristics of fitted models, the AIC values, and the benchmark concentration for 50% effect (BMC50). The model(s) with the lowest AIC value was selected.

| **model** | **No.par** | **loglik** | **AIC** | **accepted** | **BMDL** | **BMDU** | **BMD** | **conv** |
| --- | --- | --- | --- | --- | --- | --- | --- | --- |
| null | 2 | -41.56 | 87.12 |  | NA | NA | NA | NA |
| full | 8 | -13.66 | 43.32 |  | NA | NA | NA | NA |
| two.stage | 4 | -14.17 | 36.34 | yes | 4.71 | 10.8 | 7.21 | yes |
| log.logist | 4 | -14.58 | 37.16 | yes | 4.11 | 10.4 | 6.79 | yes |
| Weibull | 4 | -14.21 | 36.42 | yes | 4.51 | 10.7 | 7.15 | yes |
| log.prob | 4 | -14.52 | 37.04 | yes | 4.10 | 10.3 | 6.80 | yes |
| gamma | 4 | -14.34 | 36.68 | yes | 4.36 | 10.5 | 6.96 | yes |
| **LVM: Expon. m3-** | **4** | **-14.13** | **36.26** | **yes** | **4.69** | **10.9** | **7.41** | **yes** |
| **LVM: Hill m3-** | **4** | **-14.13** | **36.26** | **yes** | **4.69** | **10.9** | **7.40** | **yes** |

BMC50 = (7.41 +7.40)/2 = 7.41


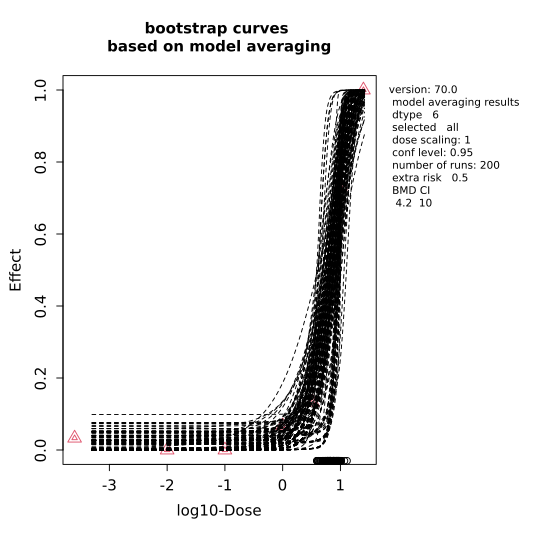

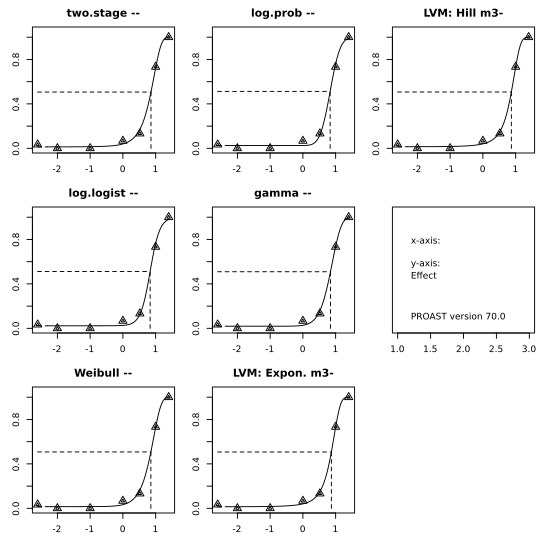


**Table. S2 (E)** Results from BMD analysis of the data on EST of SN100-DIS based on the GMS data in the ZET. The table presents characteristics of fitted models, the AIC values, and the benchmark concentration for 50% effect (BMC50). The model(s) with the lowest AIC value was selected.

| **model** | **No.par** | **loglik** | **AIC** | **accepted** | **BMDL** | **BMDU** | **BMD** | **conv** |
| --- | --- | --- | --- | --- | --- | --- | --- | --- |
| null | 2 | -46.31 | 96.62 |  | NA | NA | NA | NA |
| full | 8 | -20.52 | 57.04 |  | NA | NA | NA | NA |
| **two.stage** | **4** | **-20.88** | **49.76** | **yes** | **6.56** | **18.4** | **11.5** | **yes** |
| log.logist | 4 | -21.06 | 50.12 | yes | 5.76 | 18.5 | 10.3 | yes |
| Weibull | 4 | -20.90 | 49.80 | yes | 6.55 | 18.1 | 11.2 | yes |
| log.prob | 4 | -21.33 | 50.66 | yes | 5.63 | 19.4 | 10.2 | yes |
| gamma | 4 | -20.96 | 49.92 | yes | 6.49 | 18.0 | 10.9 | yes |
| **LVM: Expon. m3-** | **4** | **-20.88** | **49.76** | **yes** | **6.75** | **18.6** | **11.7** | **yes** |
| **LVM: Hill m3-** | **4** | **-20.88** | **49.76** | **yes** | **6.74** | **18.6** | **11.7** | **yes** |

BMC50 = (11.5 + 11.7 + 11.7)/3 = 11.63


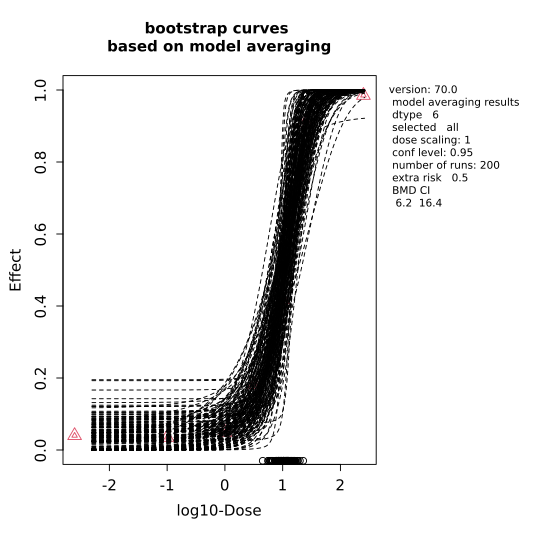

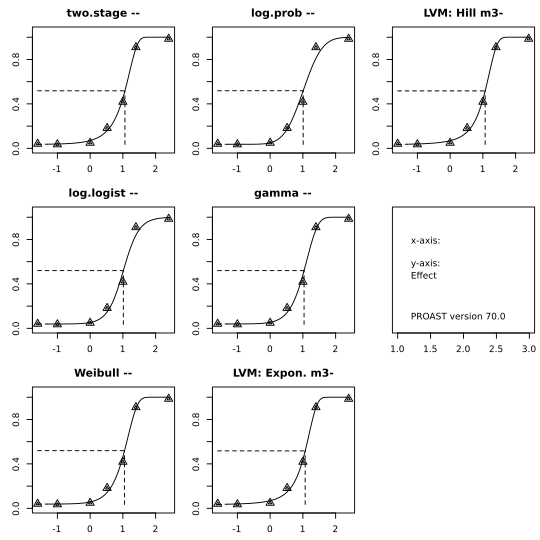


**Table. S2 (F)** Results from BMD analysis of the data on EST of SN100-AE based on the GMS data in the ZET. The table presents characteristics of fitted models, the AIC values, and the benchmark concentration for 50% effect (BMC50). The model(s) with the lowest AIC value was selected.

| **model** | **No.par** | **loglik** | **AIC** | **accepted** | **BMDL** | **BMDU** | **BMD** | **conv** |
| --- | --- | --- | --- | --- | --- | --- | --- | --- |
| null | 2 | -45.39 | 94.78 |  | NA | NA | NA | NA |
| full | 8 | -18.73 | 53.46 |  | NA | NA | NA | NA |
| **two.stage** | **4** | **-18.87** | **45.74** | **yes** | **2.53** | **7.68** | **4.63** | **yes** |
| log.logist | 4 | -19.30 | 46.60 | yes | 2.10 | 6.75 | 3.85 | yes |
| Weibull | 4 | -18.88 | 45.76 | yes | 2.40 | 7.36 | 4.40 | yes |
| log.prob | 4 | -19.23 | 46.46 | yes | 2.07 | 6.67 | 3.73 | yes |
| gamma | 4 | -18.90 | 45.80 | yes | 2.43 | 7.20 | 4.29 | yes |
| LVM: Expon. m3- | 4 | -18.94 | 45.88 | yes | 2.48 | 7.85 | 4.60 | yes |
| LVM: Hill m3- | 4 | -18.94 | 45.88 | yes | 2.47 | 7.84 | 4.53 | yes |


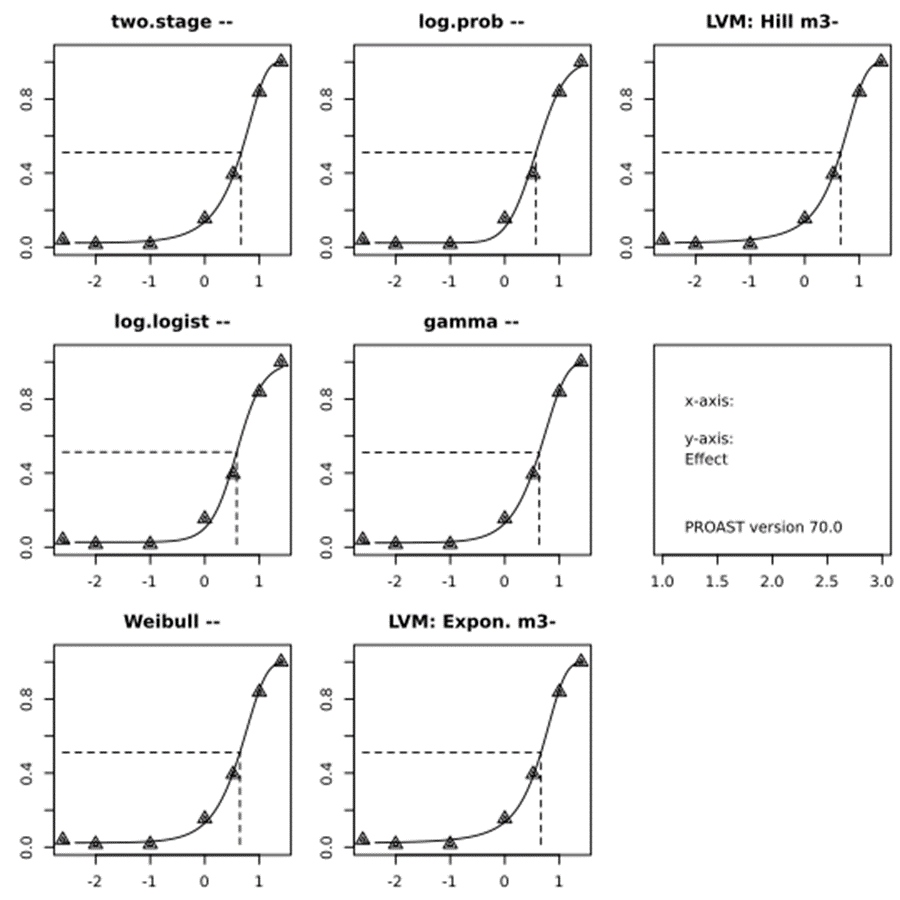

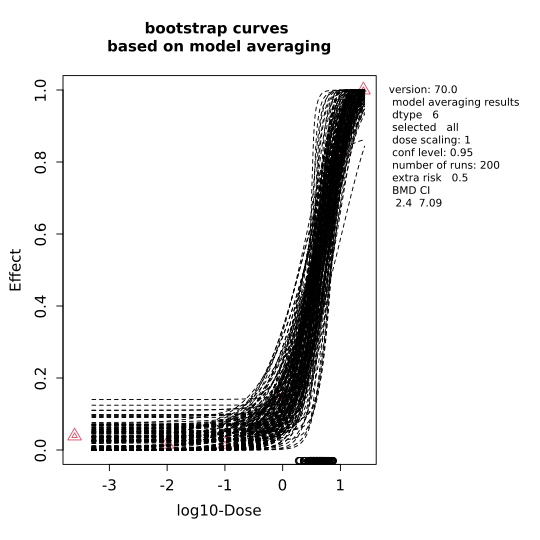


**Table. S2 (G)** Results from BMD analysis of the data on EST of SN100-DIS based on the extended-GMS data in the ZET. The table presents characteristics of fitted models, the AIC values, and the benchmark concentration for 50% effect (BMC50). The model(s) with the lowest AIC value was selected.

| **model** | **No.par** | **loglik** | **AIC** | **accepted** | **BMDL** | **BMDU** | **BMD** | **conv** |
| --- | --- | --- | --- | --- | --- | --- | --- | --- |
| null | 2 | -47.18 | 98.36 |  | NA | NA | NA | NA |
| full | 8 | -21.51 | 59.02 |  | NA | NA | NA | NA |
| **two.stage** | **4** | **-21.76** | **51.52** | **yes** | **5.41** | **16.2** | **9.93** | **yes** |
| log.logist | 4 | -21.89 | 51.78 | yes | 4.68 | 15.5 | 8.58 | yes |
| **Weibull** | **4** | **-21.76** | **51.52** | **yes** | **5.31** | **15.8** | **9.50** | **yes** |
| log.prob | 4 | -22.07 | 52.14 | yes | 4.60 | 15.9 | 8.48 | yes |
| gamma | 4 | -21.78 | 51.56 | yes | 5.34 | 15.6 | 9.25 | yes |
| LVM: Expon. m3- | 4 | -21.79 | 51.58 | yes | 5.47 | 16.5 | 9.95 | yes |
| LVM: Hill m3- | 4 | -21.79 | 51.58 | yes | 5.46 | 16.5 | 9.93 | yes |

BMC50 = (9.93+9.50)/2=9.72


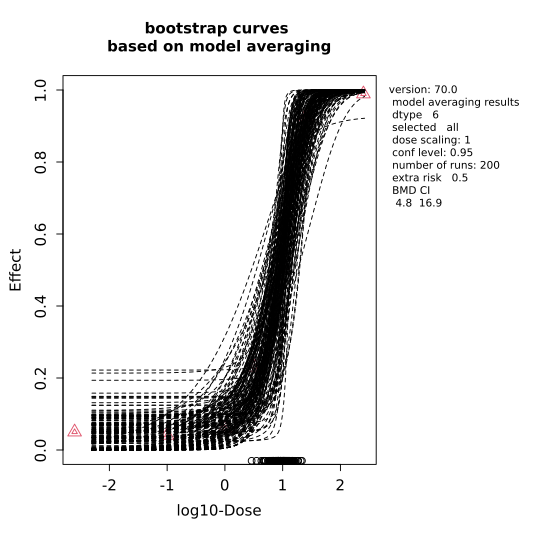

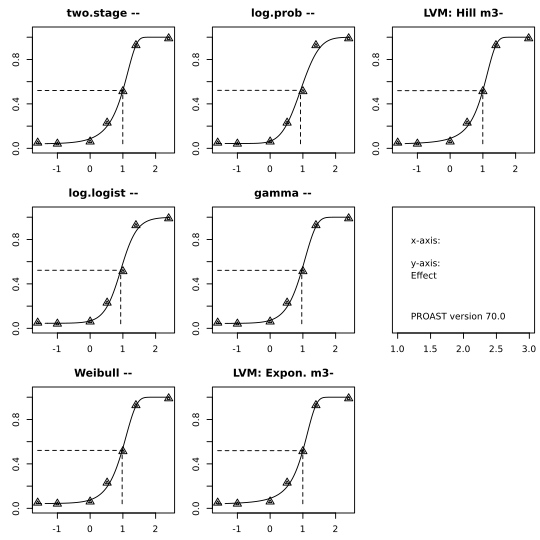


**Table. S2 (H)** Results from BMD analysis of the data on EST of SN100-AE based on the extended-GMS data in the ZET. The table presents characteristics of fitted models, the AIC values, and the benchmark concentration for 50% effect (BMC50). The model(s) with the lowest AIC value was selected.

| **model** | **No.par** | **loglik** | **AIC** | **accepted** | **BMDL** | **BMDU** | **BMD** | **conv** |
| --- | --- | --- | --- | --- | --- | --- | --- | --- |
| null | 2 | -46.26 | 96.52 |  | NA | NA | NA | NA |
| full | 8 | -19.85 | 55.70 |  | NA | NA | NA | NA |
| **two.stage** | **4** | **-20.00** | **48.00** | **yes** | **2.17** | **6.91** | **3.91** | **yes** |
| log.logist | 4 | -20.36 | 48.72 | yes | 1.72 | 5.92 | 3.29 | yes |
| Weibull | 4 | -20.01 | 48.02 | yes | 1.96 | 6.61 | 3.79 | yes |
| log.prob | 4 | -20.25 | 48.50 | yes | 1.70 | 5.82 | 3.25 | yes |
| gamma | 4 | -20.01 | 48.02 | yes | 2.03 | 6.43 | 3.73 | yes |
| LVM: Expon. m3- | 4 | -20.10 | 48.20 | yes | 2.03 | 7.03 | 3.98 | yes |
| LVM: Hill m3- | 4 | -20.10 | 48.20 | yes | 2.03 | 7.03 | 3.97 | yes |


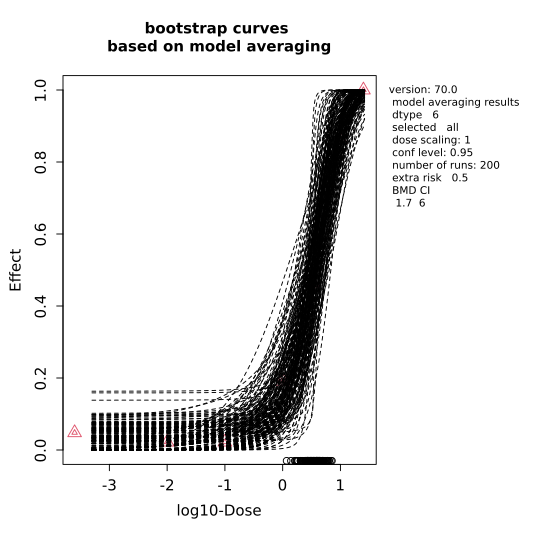

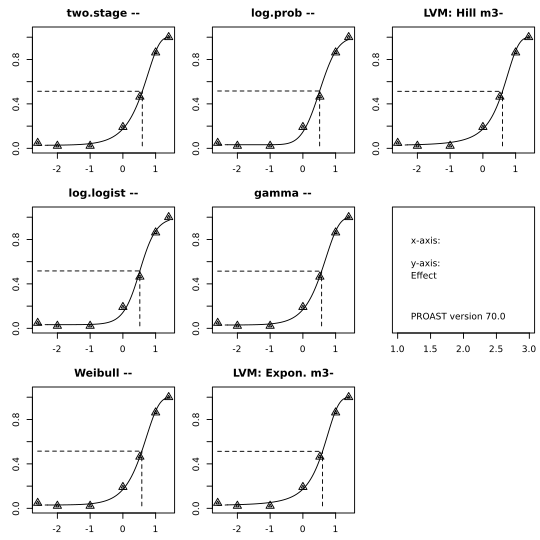


**Supplementary material 4**

Table S4. Overview of the Aromatic Ring Class (ARC) or polycyclic aromatic compound profile of petroleum substances belonging to the same product category with substances tested in the present study.

| **Substance or sample ID** | **Substance category** | **CAS no.** | **Same CAS no. with the samples tested in the present study?** | **1-ring wt.% ^1^** | **2-ring wt.%** | **3-ring wt.%** | **4-ring wt.%** | **5-ring wt.%** | **6-ring wt.%** | **≥7-ring wt.%** | **3 to 7-ring wt.%** | **Total PAC (ARC) wt.%^2^** | **IP346 (%)** |
| --- | --- | --- | --- | --- | --- | --- | --- | --- | --- | --- | --- | --- | --- |
| Site 7, Sample 23 (CRU 20906)^3^ | Distillate aromatic extract (DAE) | 64742-05-8 | Yes | 0 | 0 | 5.4 | 6.8 | 1.4 | 0 | 0 | 13.6 | 13.6 | NA |
| Site 3, Sample 13 (CRU 100709)^3^ | DAE | 64742-05-8 | Yes | 0 | 0.2 | 10.8 | 0.5 | 0 | 0 | 0 | 11.3 | 11.5 | NA |
| Site 4, Sample 3 (CRU 100711)^3^ | DAE | 64742-05-8 | Yes | 0 | 0 | 4.8 | 4.8 | 2.4 | 0.4 | 0 | 12.4 | 12.4 | NA |
| Aromatic extract (130 SUS)^4^ | DAE | 64742-04-7 | No | 0 | 0 | 2.81 | 5.72 | 2.05 | 0.11 | 0 | 10.69 | 10.8 | 19.05 |
| Aromatic extract (600 SUS)^4^ | DAE | 64742-04-7 | No | 0 | 0 | 0 | 0.29 | 1.24 | 3.07 | 2.7 | 7.3 | 7.3 | 14.34 |
| Aromatic extract 86187^4^ | DAE | 64742-04-7 | No | 0 | 0 | 4.1 | 8.1 | 6.1 | 2.0 | 0.4 | 20.7 | 20.3 | NA |
| Heavy paraffinic petroleum distillate (130 SUS)^4^ | Unrefined/acid treated oils (UATO) | 64741-51-1 | No | 0 | 0 | 1.1 | 2.38 | 0.88 | 0.04 | 0 | 4.4 | 4.4 | 9.47 |
| Heavy paraffinic petroleum distillate (600 SUS)^4^ | UATO | 64741-51-1 | No | 0 | 0 | 0 | 0.07 | 0.67 | 1.76 | 1.6 | 4.1 | 4.2 | 7.47 |
| Distillates, heavy paraffinic (sample ID 12:26)^5^ | UATO | 64741-51-1 | No | 0 | 0 | 2 | 4 | 0.7 | 0.1 | 0 | 6.8 | 6.8 | 11.4 |
| Distillates, heavy paraffinic (sample ID 12:27)^5^ | UATO | 64741-51-1 | No | 0 | 0 | 0.1 | 0.2 | 0.5 | 2 | 2 | 4.8 | 4.8 | 9 |
| Distillates, heavy paraffinic (SN600-Distillate)^6^ | UATO | 64741-51-1 | No | NA | NA | NA | NA | NA | NA | NA | NA | NA | 6.3 |
| Distillates (petroleum), solvent-refined heavy paraffinic (130 SUS)^4^ | Lubricant base oil (LBO) | 64741-88-4 | No | 0 | 0 | 0.18 | 0.21 | 0.07 | 0.01 | 0 | 0.47 | 0.48 | 0.74 |
| Distillates (petroleum), solvent-refined heavy paraffinic (600 SUS)^4^ | LBO | 64741-88-4 | No | 0 | 0 | 0.04 | 0.01 | 0 | 0.01 | 0 | 0.1 | 0.07 | 0.32 |
| Distillates (petroleum), solvent-refined heavy paraffinic ^6^ | LBO | 64741-88-4 | No | NA | NA | NA | NA | NA | NA | NA | NA | NA | 0.2 |
| Solvent-dewaxed light paraffinic (Sample ID 88088)^7^ | LBO | 64742-56-9 | Yes | 0 | 0.1 | 0.5 | 0.2 | 0.1 | 0 | 0 | 0.8 | 0.9 | NA |
| Solvent-dewaxed light paraffinic (600 SUS)^4^ | LBO | 64742-65-0 | Yes | 0.00 | 0.01 | 0.08 | 0.03 | 0.01 | 002 | 0.01 | 0.15 | 0.17 | 0.38 |
| Solvent-dewaxed light paraffinic (Sample ID 82191)^7^ | LBO | 64742-65-0 | Yes | 0 | 0.1 | 0.4 | 0.2 | 0.1 | 0.1 | 0 | 0.8 | 0.9 | NA |
| Solvent-dewaxed light paraffinic (Sample ID 86133)^7^ | LBO | 64742-65-0 | Yes | 0 | 0 | 0 | 0 | 0.1 | 0.5 | 0.5 | 1.1 | 1.1 | NA |
| Solvent-dewaxed light paraffinic (Sample ID 86142 )^7^ | LBO | 64742-65-0 | Yes | 0 | 0 | 0 | 0 | 0.1 | 0.1 | 0 | 0.2 | 0.2 | NA |
| Solvent-dewaxed light paraffinic (Sample ID 86143)^7^ | LBO | 64742-65-0 | Yes | 0 | 0 | 0.4 | 0.3 | 0.1 | 0 | 0 | 0.8 | 0.8 | NA |
| Solvent-dewaxed light paraffinic (Sample ID 86145 )^7^ | LBO | 64742-65-0 | Yes | 0 | 0 | 0 | 0.2 | 0.3 | 0.2 | 0 | 0.7 | 0.7 | NA |
| Solvent-dewaxed light paraffinic (Sample ID 86147)^7^ | LBO | 64742-65-0 | Yes | 0 | 0 | 0.1 | 0.1 | 0.2 | 0.1 | 0.2 | 0.7 | 0.7 | NA |
| Solvent-dewaxed light paraffinic (Sample ID 86149)^7^ | LBO | 64742-65-0 | Yes | 0 | 0 | 0.2 | 0.1 | 0 | 0 | 0 | 0.3 | 0.3 | NA |
| Solvent-dewaxed light paraffinic (Sample ID 87463)^7^ | LBO | 64742-65-0 | Yes | 0 | 0.1 | 0.3 | 0.3 | 0.3 | 0.3 | 0.1 | 1.3 | 1.4 | NA |
| Solvent-dewaxed light paraffinic (Sample ID 87508)^7^ | LBO | 64742-65-0 | Yes | 0 | 0 | 0.3 | 0.2 | 0 | 0 | 0 | 0.5 | 0.5 | NA |
| Solvent-dewaxed light paraffinic (Sample ID 88089)^7^ | LBO | 64742-65-0 | Yes | 0 | 0 | 0.2 | 0.2 | 0.2 | 0.1 | 0 | 0.7 | 0.7 | NA |
| Solvent-dewaxed light paraffinic (Sample ID 88090)^7^ | LBO | 64742-65-0 | Yes | 0 | 0 | 0 | 0 | 0 | 0.2 | 0.1 | 0.3 | 0.3 | NA |
| Solvent-dewaxed light paraffinic (Sample ID 88091)^7^ | LBO | 64742-65-0 | Yes | 0 | 0.3 | 0 | 0 | 0.1 | 0 | 0 | 0.1 | 0.4 | NA |
| Solvent-dewaxed light paraffinic (Sample ID 88743)^7^ | LBO | 64742-65-0 | Yes | 0 | 0 | 0 | 0 | 0 | 0 | 0.1 | 0.1 | 0.1 | NA |
| Solvent-dewaxed light paraffinic (Sample ID 88744)^7^ | LBO | 64742-65-0 | Yes | 0 | 0 | 0 | 0 | 0 | 0.1 | 0.3 | 0.4 | 0.4 | NA |
| Solvent-dewaxed light paraffinic (Sample ID 90562)^7^ | LBO | 64742-65-0 | Yes | 0 | 0 | 0 | 0.1 | 0 | 0 | 0 | 0.1 | 0.1 | NA |
| Solvent-dewaxed light paraffinic (Sample ID 90564)^7^ | LBO | 64742-65-0 | Yes | 0 | 0.2 | 0.2 | 0 | 0 | 0 | 0 | 0.2 | 0.4 | NA |
| Solvent-dewaxed light paraffinic (Sample ID 90565)^7^ | LBO | 64742-65-0 | Yes | 0 | 0 | 0 | 0 | 0.1 | 0 | 0 | 0.1 | 0.1 | NA |
| Solvent-dewaxed light paraffinic (Sample ID 91656)^7^ | LBO | 64742-65-0 | Yes | 0 | 0 | 0 | 0.4 | 0.1 | 0 | 0 | 0.5 | 0.5 | NA |
| Solvent-dewaxed light paraffinic (Sample ID 92099)^7^ | LBO | 64742-65-0 | Yes | 0 | 0 | 0.2 | 0.1 | 0 | 0 | 0 | 0.3 | 0.3 | NA |
| Solvent-dewaxed light paraffinic (Sample ID 92102)^7^ | LBO | 64742-65-0 | Yes | 0 | 0 | 0 | 0.2 | 0.2 | 0.1 | 0 | 0.5 | 0.5 | NA |
| Solvent-dewaxed heavy paraffinic (Sample ID SN600 Base oil)^6^ | LBO | 64742-65-0 | Yes | NA | NA | NA | NA | NA | NA | NA | NA | NA | 0.2 |
| Solvent-refined light paraffinic (sample ID 12:17)^5^ | LBO | 64741-89-5 | Yes | 0 | 0 | 0.3 | 0.4 | 0.1 | 0 | 0 | 0.8 | 0.7 | NA |
| Solvent-refined light paraffinic (sample ID 34:6)^5^ | LBO | 64741-89-5 | Yes | 0 | 0 | 0.1 | 0 | 0 | 0 | 0 | 0.1 | 0.1 | NA |
| Solvent-refined heavy paraffinic distillate (130 SUS)^4^ | LBO | 64741-88-4 | No | 0 | 0 | 0.18 | 0.21 | 0.07 | 0.01 | 0 | 0.47 | 0.48 | 0.2 |
| Solvent-refined heavyparaffinic distillate (600 SUS)^4^ | LBO | 64741-88-4 | No | 0 | 0 | 0.04 | 0.01 | 0 | 0.01 | 0 | 0.06 | 0.07 | 0 |
| Solvent-dewaxed light paraffinic^7^ | LBO | 64742-56-9 | Yes | 0 | 0 | 0 | 0 | 0 | 0 | 0 | 0 | 0 | NA |
| White mineral oil^7^ | Highly refined base oil (HRBO) | 8042-47-5 | Yes | 0 | 0 | 0 | 0 | 0.2 | 0 | 0 | 0.2 | 0.2 | NA |
| White mineral oil^6^ | HRBO | 64742-54-7 | No | NA | NA | NA | NA | NA | NA | NA | NA | NA | 0.20 |
| Slack wax (130 SUS)^4^ | Slack wax | 64742-61-6 | Yes | 0 | 0 | 0 | 0 | 0 | 0 | 0 | 0 | 0 | 0.11 |
| Slack wax (600 SUS)^4^ | Slack wax | 64742-61-6 | Yes | 0 | 0.06 | 0.06 | 0 | 0 | 0 | 0 | 0.06 | 0.12 | 0.18 |
| Slack wax^8^ | Slack wax | 64742-61-6 | Yes | 0 | 0 | 0 | 0 | 0 | 0 | 0 | 0 | 0 | NA |
| Slack wax (Sample ID 12:13)^9^ | Slack wax | 64742-61-6 | Yes | 0 | 0 | 0 | 0 | 0 | 0 | 0 | 0 | 0 | NA |
| Slack wax (Sample ID 23:3)^9^ | Slack wax | 64742-61-6 | Yes | 0 | 0 | 0 | 0 | 0 | 0 | 0 | 0 | 0 | NA |
| Slack wax (Sample ID 37:5)^9^ | Slack wax | 64742-61-6 | Yes | 0 | 0 | 0 | 0 | 0 | 0 | 0 | 0 | 0 | NA |
| Slack wax (Sample ID 130N 13-464)^9^ | Slack wax | 64742-61-6 | Yes | 0 | 0 | 0 | 0 | 0 | 0 | 0 | 0 | 0.1 | 0.1 |
| Slack wax (Sample ID 600N 13-467)^9^ | Slack wax | 64742-61-6 | Yes | 0 | 0.1 | 0.1 | 0 | 0 | 0 | 0 | 0.1 | 0.1 | 0.2 |
| Hard paraffin wax^8^ | Paraffin waxes and Hydrocarbon waxes | 8002-74-2 | Yes | 0 | 0 | 0 | 0 | 0 | 0 | 0 | 0 | 0 | NA |
| Highly refined hard paraffin wax^8^ | Paraffin waxes and Hydrocarbon waxes | 64742-51-4 | Yes | 0 | 0 | 0 | 0 | 0 | 0 | 0 | 0 | 0 | NA |
| Paraffin waxes and Hydrocarbon waxes^9^ | Paraffin waxes and Hydrocarbon waxes | 8002-74-2 | Yes | 0 | 0 | 0 | 0 | 0 | 0 | 0 | 0 | 0 | NA |
| Paraffin waxes (petroleum), hydrotreated^9^ | Paraffin waxes and Hydrocarbon waxes | 64742-51-4 | Yes | 0 | 0 | 0 | 0 | 0 | 0 | 0 | 0 | 0 | NA |
| Paraffin waxes and Hydrocarbon waxes, microcryst.^9^ | Paraffin waxes and Hydrocarbon waxes | 63231-60-7 | Yes | 0 | 0 | 0 | 0 | 0 | 0 | 0 | 0 | 0 | NA |

NA: Not available.

^1^1-ring wt.% is the percentage of weight of DMSO-extractable PACs that have 1 aromatic ring present in each sample, and so forth to 7 aromatic rings. Percent of each ring class was determined by Method II. Method II was developed for routine isolation, classification and quantitation of complex PAC present in petroleum fractions with boiling points ranging from >149°C to 600°C. The soluble aromatic and PACs are first extracted into cyclohexane and then extracted with DMSO. The exact procedure is described in detail in Gray et al. (2013). The obtained DMSO extracts, are first analyzed by gas chromatography with mass spectrometry (GC–MS) or flame ionization detection (FID), with naphthalene, phenanthrene, pyrene, benzo[a]pyrene, benzo[ghi]perylene and corenene standards to define the boundaries of retention times for PACs containing two- through seven-rings. After establishing the retention time markers for two through seven and greater PAC ring classes, the additional extracts are analyzed by gas chromatography with flame ionization detection (GC-FID). The results are reported as percentages of ring structures representing a ‘‘fraction of material that behaves like aromatics of the stipulated ring number’’. It should be noted that this method is not selective for low molecular weight aromatics so there may be large differences between the percentage of 1-ring structures reported in the ARC profiles and those actually present in the respective petroleum streams (Gray et al. 2013, Appendix A).

^2^ Total DMSO Extract wt.%: Percentage of weight of DMSO-extractable PACs of petroleum substances as determined by Method II.

^3^Results from High Production Volume (HPV) Chemical Challenge Program (2012): Aromatic Extracts Category Analysis and Hazard Characterization (<https://www.petroleumhpv.org/-/media/PetroleumHPV/Documents/2012_may21_Aromatic_extracts_category_final_May_18_2012.pdf>)

^4^Results from Dalbey et al (2014) Acute, Subchronic, and Developmental Toxicological Properties of Lubricating Oil Base Stocks.

^5^Results from High Production Volume (HPV) Chemical Challenge Program (2014): Lubricating base oil analytical data (<https://www.petroleumhpv.org/petroleum-substances-and-categories/lubricating-oils>)

^6^Results from Carrillo et al. (2022) Comparison of PAC and MOAH for understanding the carcinogenic and developmental toxicity potential of mineral oils

^7^Results from McKee et al (2013) Genetic toxicity of high-boiling petroleum substances.

^8^Results from HPV (2011) Waxes and Related Materials Category Analysis and Hazard Characterization (<https://www.petroleumhpv.org/-/media/PetroleumHPV/Documents/2011_01_21_Waxes_and_Related_Materials_CAD_Final.pdf>)

^9^Results from HPV (2014) Waxes Analytical data (<https://www.petroleumhpv.org/petroleum-substances-and-categories/Waxes>)
